# Insight into the Anticancer Potential of Imidazole-Based Derivatives Targeting Receptor Tyrosine Kinases

**DOI:** 10.3390/ph18121839

**Published:** 2025-12-02

**Authors:** Sami A. Al-Hussain, Dina H. Dawood, Thoraya A. Farghaly, Alaa M. Abu Alnjaa, Magdi E. A. Zaki

**Affiliations:** 1 Department of Chemistry, Faculty of Science, Imam Mohammad Ibn Saud Islamic University (IMSIU), Riyadh 11623, Saudi Arabia; 2 Chemistry of Natural and Microbial Products Department, Pharmaceutical and Drug Industries Research Institute, National Research Centre, Giza 12622, Egypt; 3 Department of Chemistry, Faculty of Science, Cairo University, Giza 12613, Egypt; 4 Department of Chemistry, Faculty of Science, Umm Al-Qura University, Makkah 21955, Saudi Arabia; 5 Department of Chemistry, Jamoum University College, Umm Al-Qura University, Makkah 21955, Saudi Arabia

**Keywords:** imidazole, benzimidazole, tyrosine kinase inhibitors, anti-cancer agents, structure-activity relationship, molecular docking

## Abstract

Kinases, which make up 20% of the druggable genome, are thought to be essential signaling enzymes. Protein phosphorylation is induced by protein kinases. Proliferation, the cell cycle, apoptosis, motility, growth, differentiation, and other biological processes are all regulated by kinases. Their dysregulation disrupts several cellular functions, leading to a variety of illnesses, the most important of which is cancer. As a result, kinases are thought to be crucial targets in a number of malignancies and other diseases. Researchers from all over the world are hard at work developing inhibitors using various chemical structures. The scaffolds of imidazole and benzimidazole provide a versatile structure for a variety of physiologically active substances. Moreover, they serve as specialized scaffolding for the creation of target-specific pharmaceuticals to address various diseases. This article seeks to illustrate the application of imidazole and benzimidazole frameworks in the formulation of inhibitors that target various tyrosine kinases, including fibroblast growth factor receptors (FGFRs), c-Met kinase, epidermal growth factor receptors (EGFRs), vascular endothelial growth factor receptors (VEGFRs), and FMS-like tyrosine kinase 3 (FLT3), from 2020 to the present. The major structure–activity correlations (SARs) of imidazole and benzimidazole derivatives were examined, and, also, a docking study highlighted the varied interactions occurring inside the active site of tyrosine protein kinases. The objective of this effort is to consolidate the fundamental structural information necessary for the synthesis of imidazole- or benzimidazole-based tyrosine kinase inhibitors with enhanced efficacy.

## 1. Introduction

Seventy-four percent of global fatalities are linked to non-communicable diseases, resulting in 41 million deaths annually [1]. In 2019, cancer ranked as the second leading cause of death, with over 18% of fatalities, whilst heart disease accounted for 33% of deaths [2]. According to the history of cancer cases, there were 10 million instances worldwide in 2000, and 6 million people died from the disease [3]. In 2018 [4], there were approximately 18.1 million illnesses and 9.6 million fatalities, in contrast to an estimated 12.7 million cases and 7.6 million deaths in 2008 [5]. In 2020, there were estimated to be 19.3 million illnesses and 10.3 million fatalities [2]. Both the number of cases of cancer and its fatalities are on the rise. Regarding cancer treatment, factors like the stage and location of the cancer influence the effectiveness of treatment. Radiation therapy, chemotherapy, and surgical radiation are among the most prevalent and traditional therapeutic methods [6,7]. Cancer prevention constitutes a key public health issue of the 21st century. According to recent data, early tumor detection may help lower the death rate from cancer [6,7].

A subclass of tyrosine kinases, termed receptor tyrosine kinases (RTKs), facilitates intercellular communication and regulates various intricate biological processes, involving cell proliferation, differentiation, motility, and metabolism. Humans possess 58 identified receptor tyrosine kinases (RTKs) [8,9], all characterized by a uniform protein structure consisting of a singular transmembrane helix, an extracellular ligand-binding domain, and an intracellular segment that includes a carboxyl (C-) terminal tail, a tyrosine kinase domain (TKD), and a juxtamembrane regulatory region [10]. Cancer is one of the numerous human diseases caused by dysregulation of RTK signaling [11].

**Figure 1:**
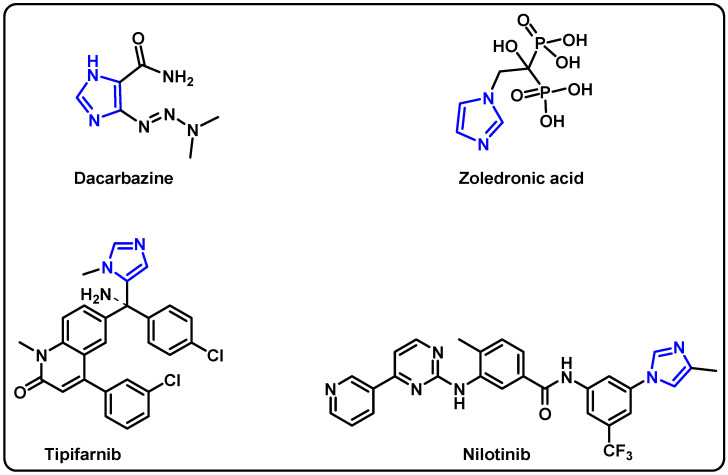
Examples of anticancer drugs containing imidazole scaffold.

Imidazole is deemed as a promising component for enhancing anticancer treatment [12,13], and several imidazole-derived medications have received FDA approval, such as dacarbazine, zoledronic acid, tipifarnib, and nilotinib; they exert their effects via distinct mechanisms of action. Nilotinib is employed in the treatment of chronic myeloid leukemia by blocking tyrosine kinase [14]. Nilotinib is acknowledged for its capacity for targeting the mast/stem cell growth factor receptor Kit (Figure 1) [15,16].

**Figure 2:**
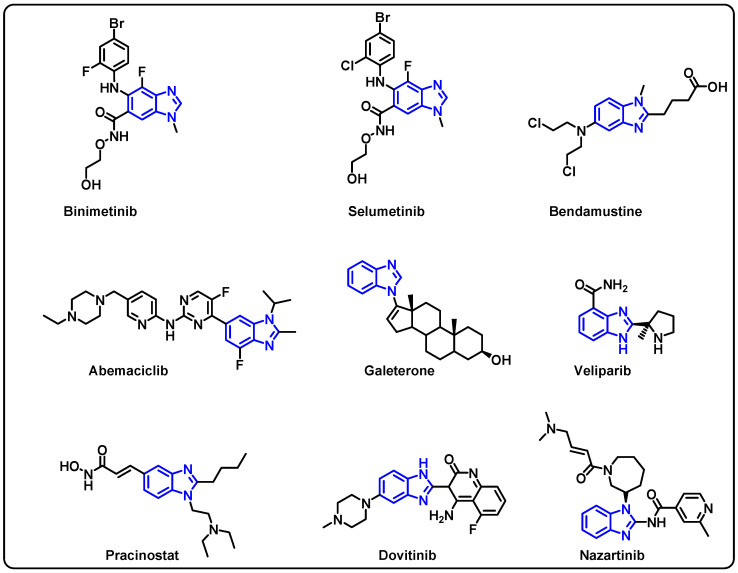
Examples of anticancer drugs containing benzimidazole scaffold.

Moreover, the benzimidazole ring system is an isostere to the guanine and adenine nucleotides, which are deemed the building units of both DNA and RNA, and this contributes to their efficient anticancer properties [17,18]. Considerable research employs the hybridization of the benzimidazole motif with diverse heterocyclic rings to identify a range of molecules aimed at eliciting various biological effects. A significant interest in benzimidazole-based compounds as anticancer medicines is attributed to its bioavailability, stability, and potency in targeting multiple receptors. Various clinically approved anticancer drugs encompass benzimidazole scaffold, as depicted in Figure 2, where Dovitinib and Nazartinib are considered as receptor tyrosine inhibitors [19]. Dovitinib is an orally bioavailable lactate salt of a benzimidazole–quinolinone hybrid identified as a multi-targeted receptor tyrosine kinase inhibitor, specifically targeting VEGFR, FLT-3, and FGFR-1 [20,21]. Dovitinib markedly suppressed lung tumor development, metastasis, and substantially extended survival in mice [22]. Dovitinib suppressed the proliferation of K562 leukemia cancer cells by functioning as an inhibitor of Topoisomerase I and II [23]. The FDA has recently granted premarket approval for Dovitinib as a companion diagnostic in patients with renal cell carcinoma (RCC). Dovitinib also halted hepatocellular carcinoma (SK-HEP1) cells in the G2/M phase, decreasing cell proliferation, obstructing bFGF-induced cell motility, and inducing apoptosis [24].

Nazartinib is a third-generation, irreversible, mutant-selective EGFR tyrosine kinase inhibitor that effectively targets the mutant EGFR T790M [25]. Nazartinib is presently undergoing phase I/II clinical trials in individuals with EGFR-mutant non-small-cell lung cancer [26].

**Figure 3:**
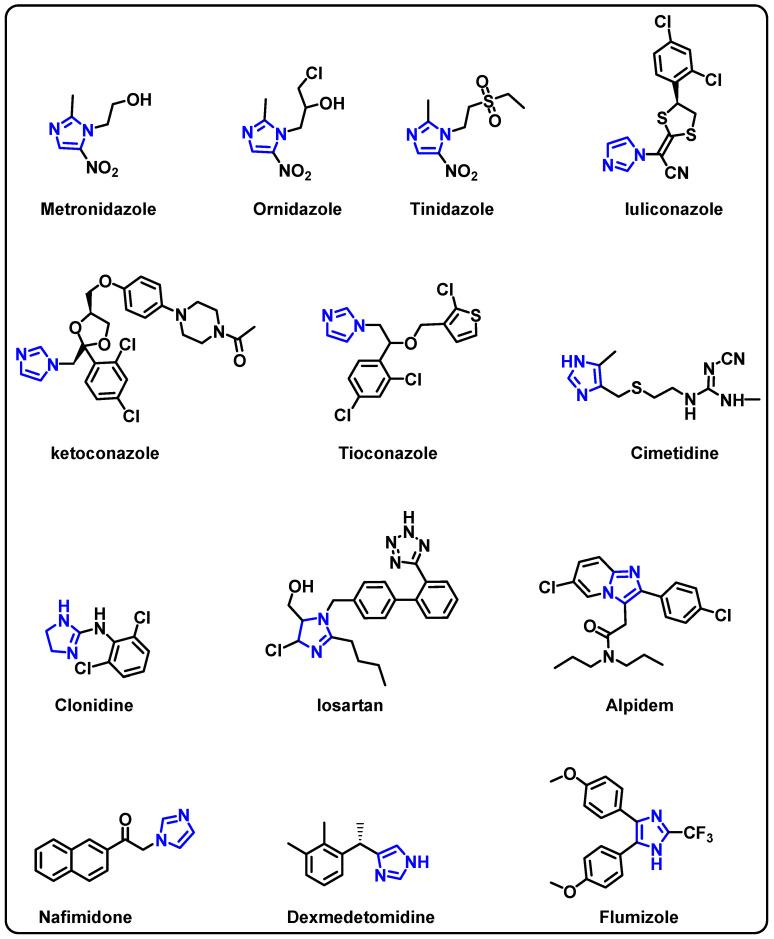
Examples of drugs containing imidazole scaffold.

**Figure 4:**
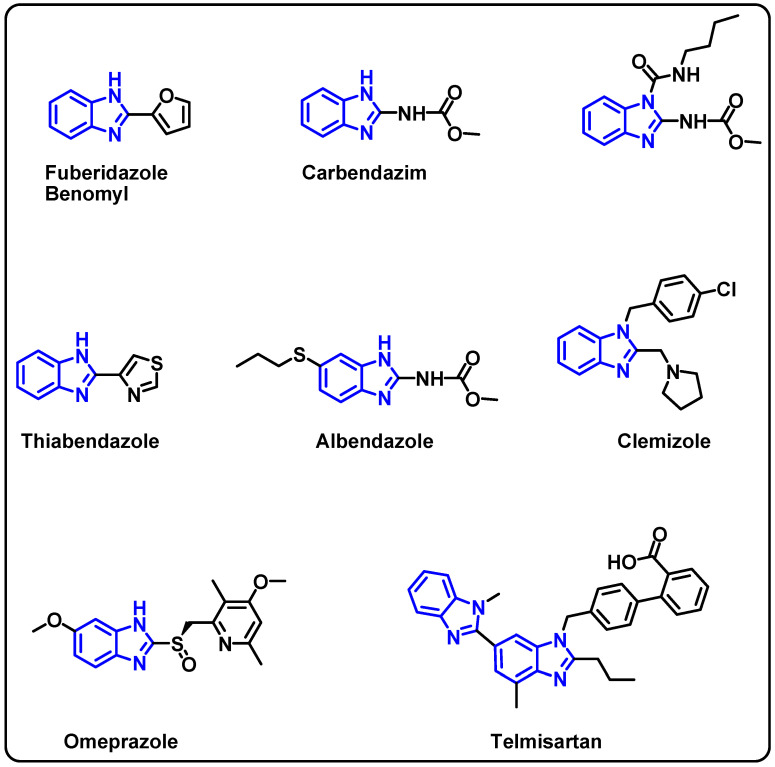
Examples of drugs containing benzimidazole scaffold.

Moreover, Figure 3 and Figure 4 illustrates various imidazole- and benzimidazole-scaffold-containing commercial drugs.

Besides receptor tyrosine kinase, diverse imidazole and benzimidazole-based derivatives presented notable inhibitory effectiveness against other kinases [27,28,29,30,31,32,33,34,35].

This review summarizes the activity of compounds containing imidazoles and benzimidazoles against various cancer cell lines targeting different receptor tyrosine kinases. The mechanism of action studies has received extensive attention, also it provides an overview of the structure–activity relationships and docking studies within the active site of varied receptor tyrosine kinases that emphasize their efficacy. The potential candidates covered in this review are categorized according to their suggested molecular target. In order to support the design and synthesis of new prominent candidates with enhanced potency and high selectivity targeting various receptor tyrosine kinases, we hope that this review of the recent literature on imidazole- and benzimidazole-based compounds as anticancer candidates, particularly as receptor tyrosine kinase inhibitors, will be helpful and motivating to researchers worldwide.

## 2. Receptor Tyrosine Kinases

### 2.1. Epidermal Growth Factor Receptor (EGFR) Inhibitors

The epidermal growth factor receptor tyrosine kinase (EGFR TK) is a member of the receptor tyrosine kinase (RTK) family, referred to ErbB. It regulates various biological processes, including angiogenesis, metastasis, apoptosis, cell motility, adhesion, and cell cycle regulation [36,37]. This enzyme is considered one of the most frequently modified oncogenes in solid tumors, such as colorectal, breast, head and neck, and non-small-cell lung cancers (NSCLCs) [38,39]. Kinase-activating mutations or EGFR overexpression induce pathological changes in EGFRs associated with adverse clinical outcomes, including early recurrence, heightened metastasis risk, and reduced survival rates [40,41,42]. Numerous small compounds serving as EGFR inhibitors are now available in the market, including the irreversible inhibitors osimertinib, dacomitinib, and afatinib, alongside the reversible inhibitors erlotinib, gefitinib, and lapatinib [43]. Prolonged administration of EGFR inhibitors, which also presents other adverse effects, results in treatment resistance [44]. Consequently, it is imperative to design and manufacture novel EGFR inhibitors that more accurately target tumor tissue and have fewer side effects.

#### 2.1.1. Imidazole-Based Derivatives as EGFR Kinase Inhibitors

**Figure 5:**
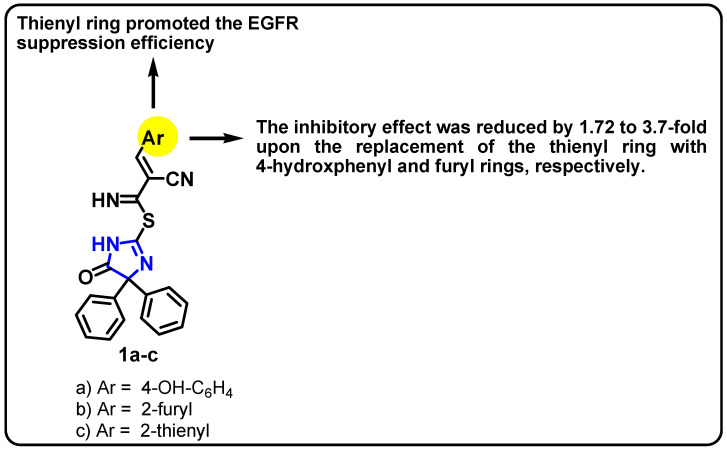
Structures of imidazole derivatives **1a–c** as EGFR kinase inhibitors.

In 2024, novel imidazolyl-2-cyanoprop-2-enimidothioate derivatives **1a–c** were recognized as effective inhibitors for EGFR, with IC_50_ equal to 0.236, 0.507, and 0.137 µM, respectively. The biological outcome disclosed that the conjugation of 2-cyanoprop-2-enimidothioate moiety with thienyl ring promoted the EGFR suppression efficiency. While the inhibitory effect was reduced by 1.72 to 3.7-fold upon the replacement of the thienyl ring with 4-hydroxphenyl and furyl rings, respectively. Furthermore, compounds **1a–c** had significant cytotoxic effects towards PC-3, MCF-7, and Hep-G2, with values of IC_50_ within low micromolar ranges of 5.35 to 27.29 µM. The most effective candidate **1c** promoted both apoptotic and necrotic mechanisms for MCF-7 cell death and paused the MCF-7 cell cycle at the S phase. It initiated apoptosis by the upregulation of caspases 8, 9, and Bax, accompanied by a downregulation of the Bcl-2 gene. Notably, the leading EGFR inhibitor **1c** exhibited a much superior docking score of -9.30 kcal/mol compared to osimertinib, the native ligand, which had an average score of −7.38 kcal/mol. The nitrile group and the side chain imino–NH engage in two hydrogen bonds with the amino acids Cys775 and Met793, respectively. Whereas arene-H interaction between Phe723 and a phenyl ring was observed (Figure 5) [45].

**Scheme 1:**
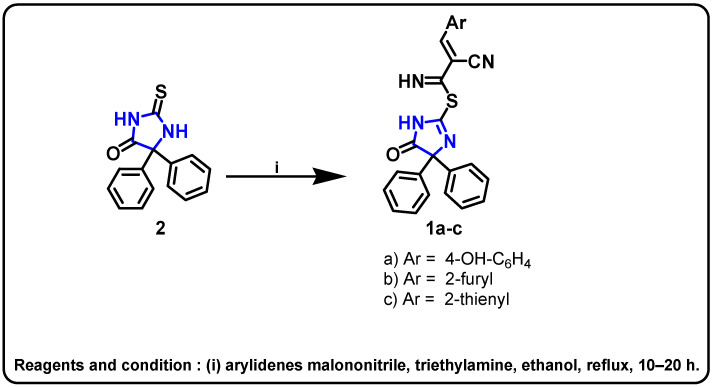
The synthesis of imidazolyl-2-cyanoprop-2-enimidothioates **1a–c**.

5,5-diphenyl-2-thioxoimidazolidin-4-one **2** reacted with arylidenes malononitrile in ethanol containing triethylamine to afford imidazolyl-2-cyanoprop-2-enimidothioates **1a–c** (Scheme 1).

**Figure 6:**
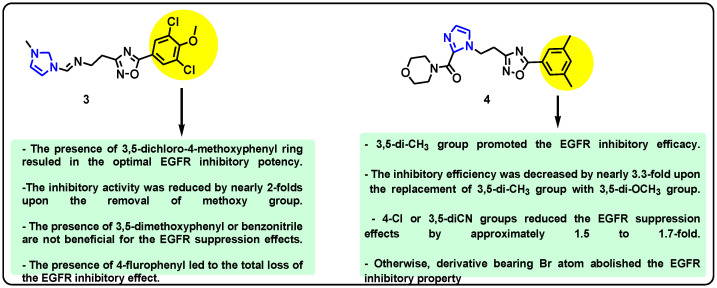
Structures of imidazole-1,2,4-oxadiazole hybrids **3** and **4** as EGFR kinase inhibitors.

A new imidazole-1,2,4-oxadiazole hybrid **3** was identified as a promising EGFR kinase inhibitor with IC_50_ = 1.21 µM. SAR analysis demonstrated that the optimal EGFR inhibitory potency was achieved via the attachment of the oxadiazole motif with 3,5-dichloro-4-methoxyphenyl ring. However, the inhibitory activity was reduced by nearly 2-fold upon the removal of methoxy group. On the other hand, the conjugation of the oxadiazole ring with 3,5-dimethoxyphenyl or benzonitrile were not beneficial for the EGFR suppression effects. Otherwise, the inhibitory effect was totally abolished upon the attachment of the oxadiazole ring with 4-flurophenyl. In addition, derivative **3** presented promising cytotoxic influence toward MCF-7, HEPG2, and A549 cancer cells with values of IC_50_ equal to 3.02, 17.11, and 23.16 µM, respectively. It was found that derivative **3** had a binding energy of −7.93 kcal/mol, which was equivalent to the binding energy of erlotinib, which was likewise docked inside the active pocket of EGFR (−7.69 kcal/mol). It formed a H-bond with Phe832 residue. In addition to this, it adhered to the guidelines established by Lipinski, Ghose, Veber, Muegge, and Egan, without any additional deviations, and it has a CLogP = 3.13 (Figure 6) [46]. Furthermore, Kannekanti et al. [47] described the synthesis and the EGFR inhibitory investigation of novel imidazole-moprholine-1,2,4-oxadiazole hybrids. Within the examined hybrids, derivative **4** displayed outstanding suppression efficacy (IC_50_ = 0.47 μM) toward EGFR comparable to the value of IC_50_ = 0.43 μM of the reference Erlotinib. The biological findings demonstrated that the EGFR inhibitory efficiency was enhanced by the connection of the oxadiazole ring to the phenyl ring, which was then substituted with an EDG such as the 3,5-di-CH_3_ group. However, the inhibitory efficiency was decreased by nearly 3.3-fold upon the replacement of the 3,5-di-CH_3_ group with a 3,5-di-OCH_3_ group. In terms of EWGs (electron-withdrawing groups) on the phenyl ring, it was discovered that the substitution of the phenyl ring with either 4-Cl or 3,5-diCN groups resulted in a reduction of the EGFR suppressing effects by roughly 1.5 to 1.7-fold. Otherwise, a derivative bearing a Br atom abolished the EGFR inhibitory property. Additionally, derivative **4** demonstrated promising anti-proliferative efficiency toward three cell lines of breast cancer, MDA-MB-231, MDA-MB-468, and MCF-7, with IC_50_ equal to 8.9, 3.7, and 4.3 µM, respectively, superior to that of the standard drug 5-Fluorouracil with IC_50_ = 10.8, 7.5, and 12.7 μM, respectively. Moreover, derivative **4** presented good binding interaction with EGFR (−9.49 kcal/mol), exceeding the score value (−7.70 kcal/mol) of erlotinib; it was involved in two H-bonds with both Met769 and Lys721 (Figure 6).

**Scheme 2:**
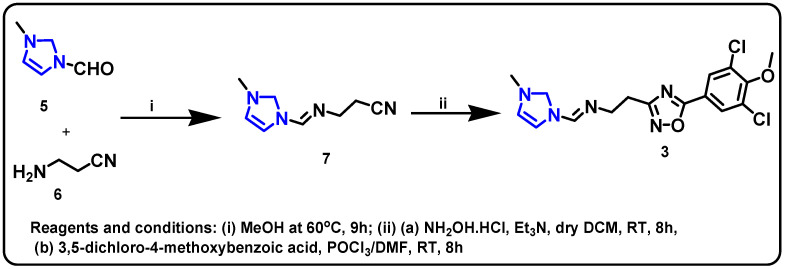
The synthesis of imidazole-1,2,4-oxadiazole hybrid **3**.

The condensation of 1-methyl-1*H*-imidazole-2-carbaldehyde **5** and 3-aminopropanenitrile **6** furnished the imine intermediate **7** that subsequently was treated with NH_2_OH.HCl to obtain the non-isolated intermediate in situ “*N′*-hydroxy-3-(1-methyl-2-oxoindolin-3-ylidene) amino) propanimidamide”, which then reacted with 3,5-dichloro-4-methoxybenzoic acid utilizing Vilsmeier reagent to obtain imidazole-1,2,4-oxadiazole hybrid **3** (Scheme 2).

**Figure 7:**
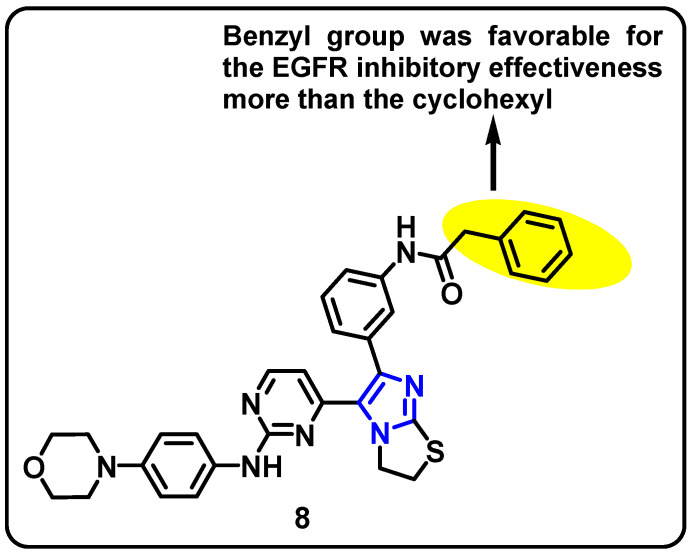
Structure of 2,3-dihydroimidazo[2,1-b]thiazole derivative **8** as EGFR kinase inhibitor.

A new 2, 3-dihydroimidazo[2,1-*b*]thiazole derivative **8** stood out as a promising EGFR kinase inhibitor, with values of IC_50_ = 35.5 and 66 nM toward the wild type and the T790M mutant form of the enzyme, respectively. Derivative **8** exhibited a drug-like profile with low elimination half-life. It was observed that the attachment of the amide moiety with the benzyl group was more favorable for the EGFR inhibitory effectiveness than the cyclohexyl (Figure 7) [48].

**Figure 8:**
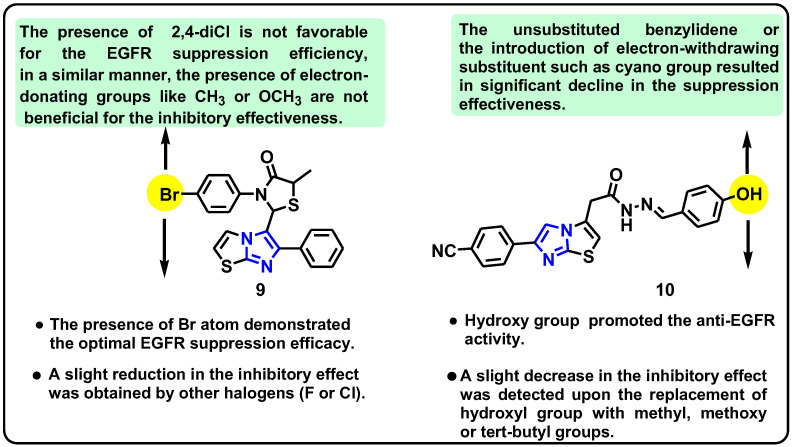
Structures of imidazothiazole–thiazolidinone hybrid **9** and imidazothiazole–hydrazone hybrid **10** as EGFR kinase inhibitors.

Furthermore, the imidazothiazole–thiazolidinone hybrid **9** was discovered by Kamboj et al. [49] as a potent EGFR inhibitor (IC_50_ = 18.35 μM). According to the results of the SAR analysis, the presence of a Br atom in the para position of the phenyl ring connected to the thiazolidinone moiety revealed the highest possible level of EGFR suppression effectiveness. Moreover, a slight reduction in the inhibitory effect was obtained by other halogens (F or Cl). However, the existence of other EWGs, such as NO_2_ or 2,4-diCl, is not beneficial to the efficiency of EGFR suppression. In a similar manner, the inclusion of an EDG, such as OCH_3_ or CH_3_, is not advantageous for the effectiveness of the inhibitory mechanism. Furthermore, it was shown that derivative **9** exhibited the highest level of activity as an anticancer candidate against MCF7, HCT116, and A549 cancer cells, with IC_50_ values of 18.73, 23.22, and 10.74 μM, respectively. It is worth noting that compound **9** exhibited an IC_50_ value of 96.38 μM when tested against the normal human embryonic kidney cell line HEK293, which serves as evidence of its safety profile. The creation of H-bonds with Cys773 and Met769, which are comparable to those formed by erlotinib, was discovered using docking analysis of derivative **9** within the EGFR active site. Other interactions with amino acid residues such as Asp831, Thr830, Lys721, and Thr766 contributed to the protein ligand complex’s increased stability (Figure 8). An experiment was conducted by Altıntop and his colleagues [50] to describe the synthesis of new imidazothiazole–hydrazone hybrids and to investigate their ability to inhibit EGFR. Among the hybrids that were investigated, derivative **10** was shown to be the most effective EGFR inhibitor, with an IC_50_ value of 9.11 µM. According to the findings of the biological investigation, the incorporation of a hydroxy group into the benzylidene moiety at the para position resulted in an increase in the suppression activity against EGFR. The substitution of methyl, methoxy, or tert-butyl groups for the hydroxyl groups resulted in a small reduction in the inhibitory effect that was observed. Otherwise, the effectiveness of the suppression was significantly reduced due to the presence of unsubstituted benzylidene or the inclusion of an EWG, such as a cyano group. Furthermore, derivative **10** demonstrated a significantly high level of cytotoxic efficacy against the A549 cell line, with an IC_50_ = 23.75 µM. The results of the flow cytometry analysis showed that derivative **10** halted the advancement of the cell cycle in A549 cells at the G2/M stage. Furthermore, it caused necrosis rather than apoptosis in the cells. Comparatively, derivative **10** has a docking score equal to −5.110 kcal/mol at the binding site of EGFR, which is significantly higher than the score of erlotinib, which was −7.150. A H-bond was formed between the NH group of the hydrazone molecule and the Asp831 residue. In light of the findings obtained through in silico analysis, derivative **10** has the potential to be regarded as drug-like molecules that possess favorable oral bioavailability (Figure 8).

**Figure 9:**
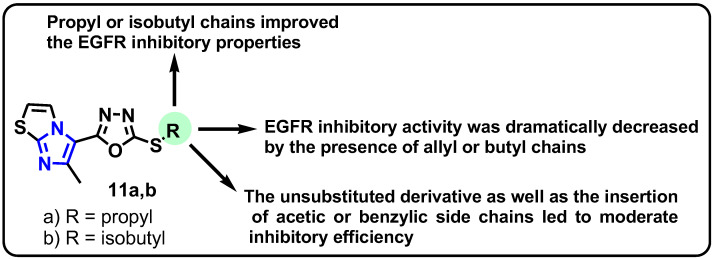
Structure of imidazothiazole–oxadiazole hybrid **11** as EGFR kinase inhibitor.

Mohammed and his colleagues [51] published a study in 2024 that described the synthesis of novel S-alkylated oxadiazole carrying imidazo[2,1-*b*]thiazole derivatives and its inhibitory assessment against EGFR kinase. Among the candidates that were evaluated, derivatives **11a** and **11b** exhibited remarkable suppression activity (IC_50_ = 0.099 and 0.086 μM, respectively), which was comparable to the standard medication erlotinib (IC_50_ = 0.046 μM). According to the findings of the SAR analysis, the inhibitory impact of EGFR was enhanced in derivatives that were alkylated with aliphatic side chain substituents such as propyl or isobutyl chains. On the other hand, the presence of allyl or butyl chains resulted in a significant reduction in the inhibitory activity. On the other hand, the unsubstituted derivative, as well as the insertion of acetic or benzylic side chains, led to moderate inhibitory efficiency. Furthermore, it is worth noting that derivatives **11a** and **11b** exhibited outstanding cytotoxic efficacy (IC_50_ = 2.27 and 1.46 μM, respectively) when it came to MCF-7 and MDA-MB-468 cell lines. This was in comparison with erlotinib, which exhibited a cytotoxic efficacy of 7.82 and 10.13 μM, respectively. Furthermore, it was observed that derivatives **11a** and **11b** exhibited the most promising safety profiles when tested against the normal MCF-10a breast cell line (IC_50_ = 26.66 and 41.31 μM, respectively) in comparison to erlotinib (IC_50_ = 16.32 μM). A flow cytometric investigation revealed that derivatives **11a** and **11b** halted the cell cycle during the S phase in MCF-7 and MDB-MB-468, respectively. In the sarcoma mice model, the results of the in vivo biodistribution of ^99m^Tc-**11b** complex verified the potential of derivative **11b** to target tumor cells in vivo. This was demonstrated by a ratio of around seven between the target (sarcoma muscle) and the non-target (normal muscle). Within the EGFR active site, the binding mechanism of derivatives **11a** and **11b** revealed the development of a H-bond acceptor between the N7 position of the imidazothiazole scaffold and Met769. Another H-bond that was bridged through a water molecule was found to exist between the oxadiazole ring’s oxygen and Thr766, whilst the alkyl substituents are involved in hydrophobic interactions with Lys721, Glu738, Leu764, Ile765, and Met742 (Figure 9).

**Figure 10:**
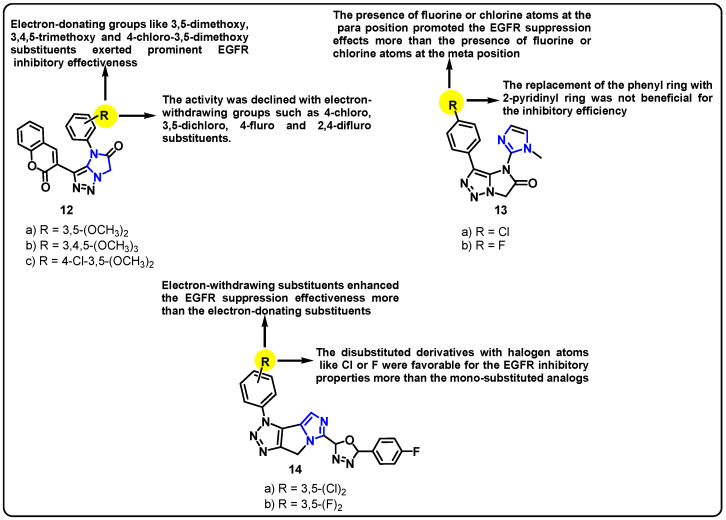
Structures of coumarine-imidazo[1,2-c][1,2,3] triazoles **12a**–**c,** imidazole-imidazo[1,2 c][1,2,3] triazoles **13a**,**b,** and 1,3,4-oxadiazole-imidazo[1′,5′:1,2]pyrrolo[3,4-d][1,2,3]triazoles **14a**,**b** as EGFR kinase inhibitors.

Samala and colleagues [52] demonstrated the synthesis of novel coumarine-imidazo[1,2-*c*][1,2,3] triazoles and examined their capacity to inhibit EGFR kinase. Among the evaluated candidates, compounds **12a**–**c** emerged as the most potent EGFR inhibitors (IC_50_ = 0.420, 0.367, and 0.453 μM, respectively) in comparison to Erlotinib (IC_50_ = 0.460 μM). SAR analysis revealed that compounds containing a phenyl ring substituted with EDG such as 3,5-dimethoxy, 3,4,5-trimethoxy, and 4-chloro-3,5-dimethoxy had significant EGFR inhibitory potency. However, the activity declined with derivatives bearing a phenyl ring substituted with EWGs such as 4-chloro, 3,5-dichloro, 4-fluro, and 2,4-difluro substituents. Moreover, derivatives **12a**–**c** demonstrated prominent cytotoxic efficiency against the A-549 (IC_50_ = 5.31, 3.54, and 6.57 μM, respectively), surpassing that of Erlotinib (IC_50_ = 10.12 μM) (Figure 10). According to the findings of the binding interactions between the effective inhibitors and the EGFR kinase, derivatives **12a**–**c** exhibited considerable binding energies (−8.98, −8.55, and −9.08 kcal/mol, respectively) that were higher than the binding energy of Erlotinib (−7.69 kcal/mol). In the in silico pharmacokinetic profile (ADMET), it was proven that the water solubility (log S) of the derivatives **12a**–**c** varied among −3.462 to −3.626, while the Caco2 permeability varied from 0.461 to 1.125. Furthermore, they disclosed 100% intestinal absorption. None of the candidates that were evaluated, on the other hand, exhibited permeability to the BBB or the CNS. Aside from that, each and every one of them exhibited interactions with cytochrome P450 and inhibited CYF1A2. It was determined through the toxicity prediction that each and every one of them was hepatotoxic and inhibited hERG II functions. It was found that the lipophilicity of the compounds (ClogP) varied from 2.28 to 2.85. In 2024, it was revealed that fused imidazole-imidazo[1,2-*c*][1,2,3]triazoles **13a,b** had promising effectiveness as EGFR inhibitors. Their IC_50_ values were 0.38 and 0.42 µM, respectively, which were comparable to the value of IC_50_ of erlotinib, which was 0.42 µM. As a result of the biological findings, it was determined that the presence of fluorine or chlorine atoms at the *para* position on the phenyl ring connected to the triazole moiety was more effective in suppressing the effects of EGFR than the presence of fluorine or chlorine atoms at the *meta* position on the phenyl ring. Furthermore, the substitution of the phenyl ring with the 2-pyridinyl ring did not result in any improvement to the inhibitory efficacy. Furthermore, derivatives **13a** and **13b** exhibited significant in vitro cytotoxic activity against two breast cancer cell lines, namely, MCF-7 and MDA-MB231. The IC_50_ values for **13a** were 4.02 and 6.92 µM, respectively, while the IC_50_ values for **13b** were 4.23 and 6.93 µM, respectively. This is in contrast to the behavior of erlotinib, which exhibited IC_50_ values of 4.70 and 7.21 µM, respectively. In the course of docking simulations carried out on the human EGFR TKD protein, it was discovered that derivative **13a** exhibited superior binding affinity (−7.86 Kcal/mol) and interaction patterns. A H-bond was detected between the nitrogen atom of the triazole moiety and Met769. An alkyl interaction was established between Leu820 and the methyl group linked to the imidazole ring. Additionally, pi–alkyl interactions were observed between the amino acids Leu820, Leu694, Val702, and Ala719 and the aromatic heterocyclic moieties. Furthermore, Gly772 and Cys773 established a carbon–hydrogen bond and a pi-donor hydrogen bond, respectively (Figure 10) [53]. The same group of researchers [54] published the synthesis and anti-EGFR assessment of novel 1,3,4-oxadiazole-imidazo[1′,5′:1,2]pyrrolo[3,4-*d*][1,2,3]triazole derivatives. Among the evaluated candidates, derivatives **14a** and **14b** exhibited remarkable EGFR inhibition, with IC_50_ = 0.29 and 0.37 μM, respectively, surpassing the IC_50_ = 0.44 μM of Erlotinib. SAR analysis pointed out that analogs with EWGs on the fused triazole ring improved EGFR suppression efficacy more than those with EDGs. Moreover, the disubstituted derivatives containing halogen atoms such as Cl or F exhibited superior EGFR inhibitory capabilities compared to the mono-substituted analogs. Additionally, derivatives **14a** and **14b** exhibited significant in vitro cytotoxic efficacy against two cell lines of human lung cancer, A-549 and NCI-H460, with IC_50_ values between 3.46 and 5.43 μM. Moreover, at doses of 15 and 20 μg/mL, the most effective derivatives **14a** and **14b** markedly activated caspases 3/7, 8, and 9. The docking simulation performed on the human EGFR TKD protein indicated that derivative **14b** had the maximum binding energy of −7.4 Kcal/mol. Derivative **14a** had a binding affinity of −7.0 Kcal/mol. Both derivatives established hydrogen bond connections between the nitrogen atoms of the triazole and oxadiazole heterocyclic rings with Met769 and Lys721, respectively. The core tricyclic ring exhibited an interaction of π-sigma with VAL702, in addition to interactions of carbon-H bond with Leu698. Furthermore, the residues Ala719, Leu764, Leu694, and Leu820 exhibited alkyl and π-alkyl interactions (Figure 10).

**Figure 11:**
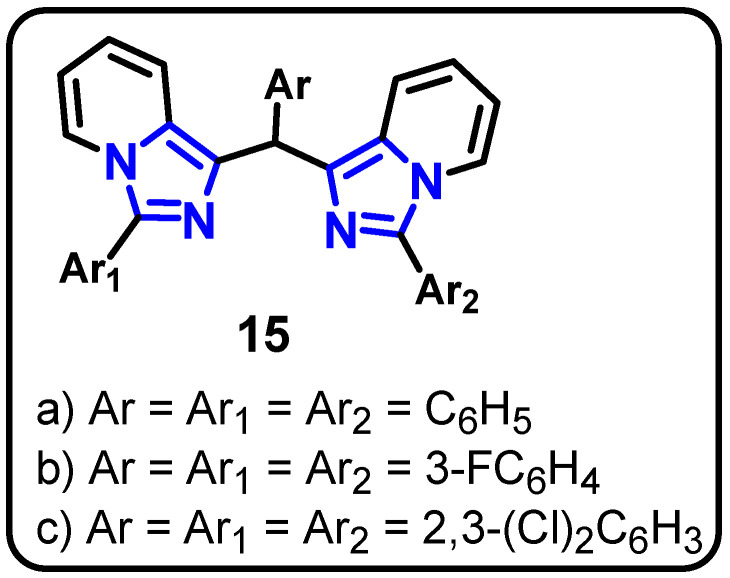
Structures of imidazole[1,5-a]pyridine derivatives **15a**–**c** as EGFR kinase inhibitors.

In a theoretical investigation, a series of 18 derivatives of fused imidazolepyridine were synthesized, and their efficacy was assessed utilizing a realistic computational drug design scenario. Three derivatives, **15a**–**c**, were identified as effective EGFR inhibitors based on theoretical evidence, including significant rupture force and substantial binding free energy. Derivative **15a** exhibited the most substantial binding free energy of −12.8 kcal/mol, markedly surpassing that of osimertinib and erlotinib, which were −8.7 and −9.1 kcal/mol, respectively. Consequently, derivative **15a** was identified as the most outstanding candidate for EGFR inhibition. Derivatives **15b** and **15c** have values of −11.7 and −10.6 kcal/mol, respectively, equivalent to erlotinib and osimertinib (Figure 11). The three compounds exhibited significant potential as prospective anticancer agents [55].

**Figure 12:**
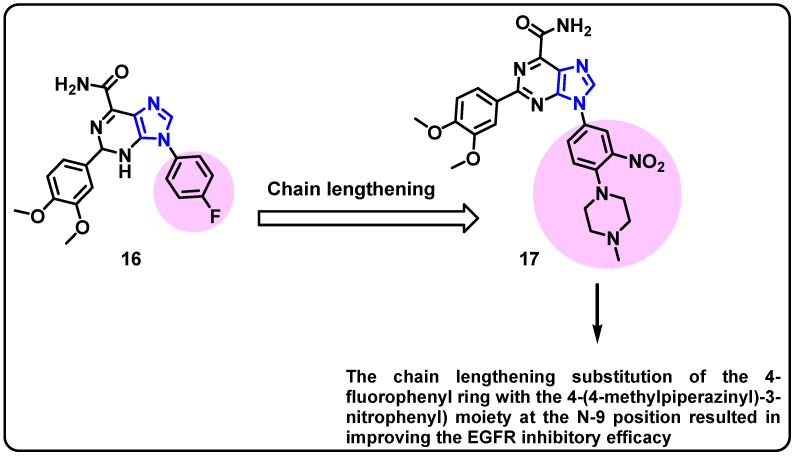
Structures of fused imidazole derivatives **16** and **17** as EGFR kinase inhibitors.

New fused imidazole derivatives **16** and **17** emerged as potent EGFR inhibitors with IC_50_ = 617.33 and 236.38 nM, respectively, relative to the IC_50_ = 239.91 nM of erlotinib. It was noticed that the substitution of the ring of 4-fluorophenyl with the long chain of the 4-(4-methylpiperazinyl)-3-nitrophenyl) moiety at the N-9 position resulted in improving the EGFR inhibitory efficacy. Additionally, derivative **16** was displayed as a promising anticancer molecule against cancer cell lines, viz., MDA-MB-231, T47D, A549, and MCF-7, with values of IC_50_ ranging from 2.29 to 9.96 µM. However, derivative **17** demonstrated a great amelioration in anticancer efficiency, showing IC_50_ values at extremely low micromolar concentrations ranging from 1.98 to 4.07 μM. Moreover, derivative **17** halted the cell cycle growth in MDA-MB-231 cells at the sub-G1 phase. As expected, the binding dock score of derivative **17** (dock score = −7.545) was higher than that of its corresponding parent derivative **16** (R isomer) (dock score = −6.227). Derivative **17** created a H-bond between CONH_2_ and Met769. Whereas the *N*-methyl piperazinyl moiety was located adjacent to the DFG motif (Gly833, Asp831, and Phe832), whereas the NO_2_ group was noted to engage with Asp831 (Figure 12) [56].

**Figure 13:**
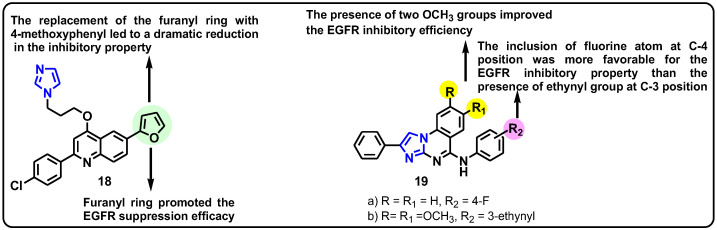
Structures of imidazole–quinoline hybrid **18** and imidazo[1,2-a]quinazoline derivatives **19a**,**b** as EGFR kinase inhibitors.

The new imidazole–quinoline hybrid **18** emerged as a prominent EGFR inhibitor with IC_50_ equal to 33.65 nM, exceeding that of the utilized reference Gefitinib (IC_50_ = 48.52 nM). SAR investigation displayed that the quinolone scaffold, when linked at the 6th position with a furanyl ring, promoted EGFR suppression efficacy. Whereas the replacement of the furanyl ring with 4-methoxyphenyl led to a dramatic reduction in the inhibitory property. Furthermore, derivative **18** presented remarkable cytotoxic efficacy toward each of HCT-116 and DLD1, the cell lines of colorectal cancer (IC_50_ = 4.75 and 4.46 µM, respectively), which was almost 1.46 to 2.29-fold more efficient than Gefitinib (IC_50_ = 6.94 and 10.24 µM, respectively). The imidazole ring is engaged in two H-bonds with Lys721 and Thr766 amino acids. Moreover, hydrophobic interactions were involved between the quinolone scaffold with Thr830 and Val 702 as well as with Leu820 and Leu694. Additionally, pi–pi stacking was detected between the furan moiety and Phe699 (Figure 13) [57]. Hasanvand et al. [58] documented a method for the synthesis and evaluation of novel imidazo[1,2-a]quinazoline derivatives targeting EGFR kinase. The Kinase assay demonstrated that compounds **19a** and **19b** exhibited inhibitory efficacy and selectivity towards EGFR, with IC_50_ = 82.0 and 12.3 μM, respectively. Furthermore, they inhibited the phosphorylation of EGFR and its downstream effector (ERK1/2). The biological results demonstrated that the addition of two groups of OCH_3_ at positions C-7 and C-8 of imidazo[1,2-*a*]quinazoline improve the EGFR inhibitory efficiency. The incorporation of a fluorine atom at the C-4 position of the aniline moiety was more advantageous for the EGFR inhibitory function than the presence of an ethynyl group at the C-3 position. Moreover, derivatives **19a** and **19b** exhibited encouraging anti-proliferative effects, with values of IC_50_ between 0.04 and 18.86 μM toward HepG2, HeLa, PC3, and MDA-MB-231, compared to Erlotinib. Additionally, they demonstrated limited cytotoxic efficacy against a normal lung cell line (MRC5), with IC_50_ = 61.81 and 67.54 μM, respectively. They also induced apoptosis and G0 cell cycle arrest in PC3 and HeLa cells. Derivatives **19a** and **19b** exhibited favorable docking scores of −8.22 and −9.70 kcal/mol, respectively, comparable to Erlotinib’s value of −8.57 kcal/mol. Derivative **19a** formed one hydrogen bond with Thr766 and engaged in many hydrophobic contacts with Thr766, Leu820, Leu694, Ala719, Lys721, and Leu768. Conversely, derivative **19b** established four H-bonds with the residues Cys773, Gly772, Met769, and Thr766. Additionally, it participated in many hydrophobic interactions with the amino acid residues Leu768, Thr766, Ala719, Leu694, Leu820, Lys704, Lys721, and Leu764 (Figure 13).

#### 2.1.2. Benzimidazole-Based Derivatives as EGFR Kinase Inhibitors

**Figure 14:**
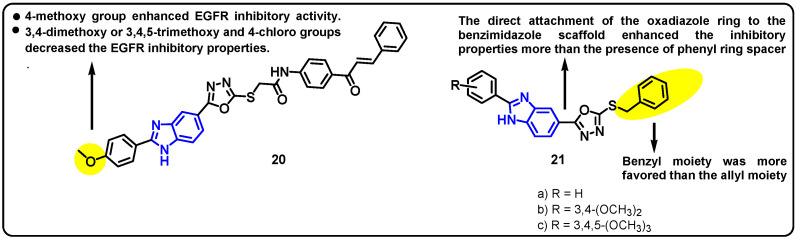
Structures of benzimidazole–oxadiazole hybrids **20** and **21** as EGFR kinase inhibitors.

In the year 2023, novel hybrids consisting of benzimidazole, oxadiazole, and chalcone were synthesized and examined for their ability to inhibit EGFR kinase. Among the investigated hybrids, derivative **20** was the most efficient inhibitor, with a value of IC_50_ = 0.55 µM, compared to erlotinib (IC_50_ = 0.08 µM). Derivative **20** also presented remarkable anticancer efficiency toward Panc-1, A549, and MCF-7 cell lines (IC_50_ = 1.30, 1.20, and 0.95 µM, respectively), which was equipotent to that of Doxorubicin (IC_50_ = 1.41, 1.21, and 0.90 µM, respectively). It triggered apoptosis and arrested cell cycle of MCF-7 at G1/S; also, it raised the Bax level and declined the Bcl-2 level similarly to Doxorubicin. The docking study revealed the formation of two H-bonds between the NH of benzimidazole and the C=O of the chalcone moiety with Asp831 and Met769, respectively. In addition to hydrophobic interactions with Leu834, Phe699, Val702, and Leu820. Interestingly, compound **20** revealed an ADMET profile similar to erlotinib. SAR analysis concluded that substituting the phenyl moiety on the benzimidazole ring with a 4-methoxy group significantly improved EGFR suppression efficacy compared to the unsubstituted phenyl or substitutions with 3,4-(OCH_3_)_2_ and 3,4,5-(OCH_3_)_3_ groups. A notable reduction in inhibitory characteristics was observed when the 4-methoxy group was exchanged with an electron-withdrawing moiety, such as a 4-chloro atom (Figure 14) [59]. After two years, the same researchers [60] synthesized and assessed new benzimidazole–oxadiazole hybrids against EGFR and BRAF^V600E^. Among the screened hybrids, derivatives **21a**–**c** demonstrated remarkable inhibitory effects against EGFR (IC_50_ = 61, 67, and 63 nM, respectively) and BRAF^V600E^ (IC_50_ = 43, 49, and 51 nM, respectively) comparable to that of Erlotinib (IC_50_ = 80 nM) and Vemurafenib (IC_50_ = 30 nM). The SAR research indicated that the benzyl group was preferred over the allyl group for the suppression actions of both EGFR and BRAFV600E. Furthermore, the direct attachment of the oxadiazole ring to the benzimidazole scaffold improved the inhibitory properties more than the presence of phenyl ring spacer. The unsubstituted phenyl ring attached to the benzimidazole improved the inhibitory effectiveness compared to the substituted phenyl rings. In addition, derivatives **21a**–**c** revealed significant anti-proliferative efficiency against HT-29, MCF-7, Panc-1, and A-549, with values of IC_50_ ranging between 24 nM and 32 nM. Notably, derivative **21a** triggered apoptosis in both early and late phases, stopped cells of A-549 in the phase of G0/G1, and suppressed the progression from the G1 phase to the S and G2/M stages. A docking study of **21a** against EGFR got a good score (−11.84 kcal/mol). Hydrophobic interactions were detected between compound **21a** with Phe771, Leu694, Leu768, phe699, LEU-820, and Val702 amino acid residues (Figure 14).

**Figure 15:**
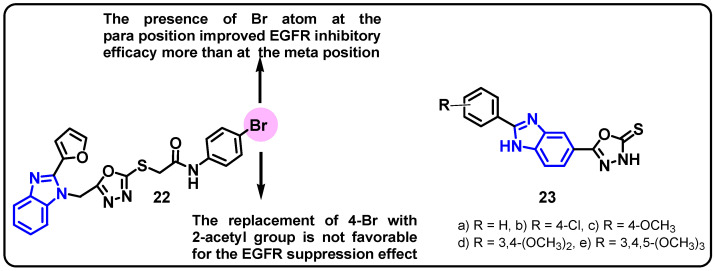
Structures of benzimidazole–oxadiazole hybrids **22** and **23a-e** as EGFR kinase inhibitors.

A new benzimidazole–oxadiazole hybrid bearing the thioacetamide linker **22** emerged as a promising EGFR inhibitor with IC_50_ equal to 0.91 µM. A SAR study displayed that the existence of a Br atom on the phenyl ring at the *para* position improved EGFR inhibitory efficacy more than its presence at the *meta* position. The replacement of 4-Br with a 2-acetyl group is not conducive to the efficacy of EGFR inhibition. Furthermore, derivative **22** demonstrated outstanding anti-proliferative potency toward HepG2 and MCF-7 (IC_50_ = 8.92 and 3.56 µM, respectively) in comparison to tamoxifen (IC_50_ = 21.76 and 18.94 µM, respectively). Otherwise, it displayed a weak cytotoxic effect on MRC-5 (cells of normal human lung fibroblast), with an IC_50_ = 221.19 µM (Figure 15) [61]. In 2023, Hagar and her colleges [62] developed and synthesized novel hybrids of benzimidazole and oxadiazole **23a**–**e** as inhibitors of EGFR (Figure 15). The produced hybrids exhibited strong binding affinity at the EGFR binding site. The docking scores of the hybrids varied between −7.6 kcal/mol and −8.7 kcal/mol, which were comparable to Erlotinib’s value of −9.7 kcal/mol. The critical amino acid residues Leu820, Leu694, Lys721, and Val702 participate in hydrophobic interactions with benzimidazole–oxadiazole hybrids **23a**–**e**. The introduction of an EDG on the phenyl moiety led to diminished docking scores.

**Figure 16:**
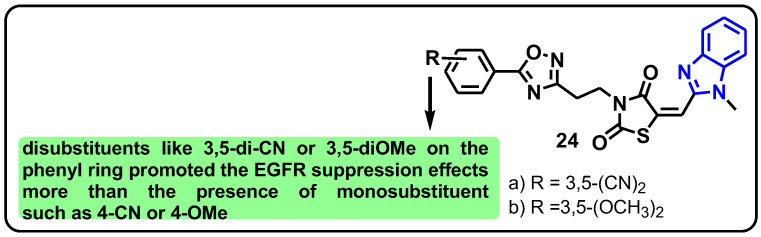
Structures of hybrids bearing benzimidazole, thiazolidine-2,4-dione, and 1,2,4-oxadiazole scaffolds **24a**,**b** as EGFR kinase inhibitors.

New hybrids bearing 1,2,4-oxadiazole, thiazolidine-2,4-dione, and benzimidazole frameworks **24a** and **24b** emerged as eminent EGFR inhibitors, exhibiting IC_50_ values of 0.26 and 0.23 µM, respectively, surpassing that of Erlotinib (IC_50_ = 0.4 µM). The biological data concluded that the presence of disubstituents like 3,5-di-CN or 3,5-diOMe on the phenyl ring promoted the EGFR suppression effects more than the presence of a monosubstituent such as 4-CN or 4-OMe. Furthermore, derivative **24a** demonstrated superior anti-proliferative efficiency toward A549, MCF-7, and HepG2 (IC_50_ = 19.72, 1.32, and 11.27 µM, respectively) as compared to Erlotinib (IC_50_ = 20.10, 4.15, and 13.30 µM, respectively). Derivative **24a** displayed the greatest binding energy (−10.92 kcal/mol). Derivative **24a** established two H-bonds with Asp831 and Lys721, along with pi–cation interactions involving Arg817 and Lys721 residues (Figure 16) [63].

**Figure 17:**
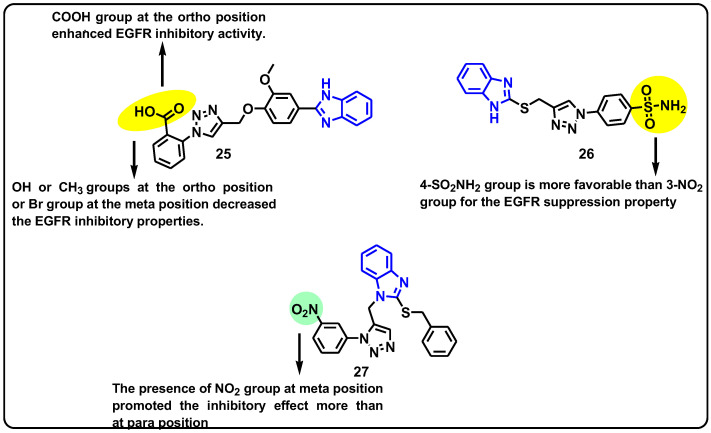
Structures of benzimidazole–triazole hybrids **25**–**27** as EGFR kinase inhibitors.

Alzahrani and his colleagues [64] identified a novel benzimidazole–triazole hybrid **25** as an effective EGFR inhibitor, with an IC_50_ value of 0.52 μM, in comparison to erlotinib’s IC_50_ of 0.41 μM. Compound **25** exhibited remarkable anti-proliferative activity against HCT-116, HepG-2, and MCF-7 cell lines, with IC_50_ values of 2.35, 1.85, and 1.49 µM, respectively, comparable to doxorubicin, which had IC_50_ values of 2.12, 1.82, and 1.45 µM, respectively. SAR analysis disclosed that the existence of a COOH group at the *ortho* position of the phenyl moiety linked to the triazole ring was the most significant EGFR inhibitor. The inclusion of OH or CH_3_ groups at the *ortho* location, or a bulky group at the *meta* position such as Br, diminished the EGFR inhibitory characteristics by approximately 1.77 to 5.15-fold (Figure 17). Moreover, the novel benzimidazole–triazole hybrids **26** and **27** demonstrated significant EGFR inhibitory activity, with IC_50_ values equal to 78 and 73 nM, respectively, surpassing that of erlotinib (IC_50_ = 80 nM). Regarding derivative **26**, the biological data indicated that substituting the phenyl ring linked to the triazole moiety with a 4-SO_2_NH_2_ group is more advantageous than using a 3-NO_2_ group for EGFR suppression efficacy. In derivative **27**, the existence of the NO_2_ substituent at the meta position on the phenyl moiety linked to the triazole moiety enhanced inhibitory efficacy more than the *para* position. Otherwise, the exchanging of the 3-NO_2_ group with 4-OCH_3_ resulted in reducing EGFR inhibitory action. Derivatives **26** and **27** were recognized as the most significant anti-proliferative candidates, exhibiting values of GI_50_ = 29 and 25 nM, respectively. They stimulated apoptosis through activating Bax, caspase-3, and caspase-8, while simultaneously downregulating the anti-apoptotic protein Bcl-2. The docking analysis of derivative **26** at the EGFR binding site demonstrated the establishment of a hydrogen bond between the nitrogen of the triazole ring and Met769. A further hydrogen bond was identified between the amino sulfonyl moiety and Leu694. Derivative **27** demonstrated the establishment of a water-mediated hydrogen bond between the nitro group and Pro770. Furthermore, the sulfur atom established an extra hydrogen bond with Leu820. The ADME assessment of these hybrids underscores their efficacy as potential therapeutic agents (Figure 17) [65].

**Figure 18:**
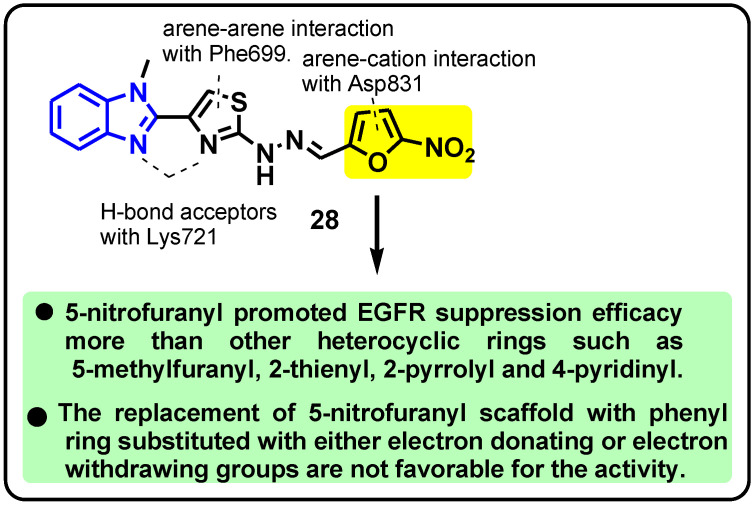
Structure of benzimidazole–thiazole hybrid **28** as EGFR kinase inhibitor.

In 2020, Srour et al. [66] discovered a new benzimidazole–thiazole hybrid **28** as a prominent EGFR inhibitor. It displayed two-fold more efficient suppression effectiveness against EGFR with its IC_50_ = 71.67 nM compared with the IC_50_ = 152.59 nM of erlotinib. It also displayed good cytotoxic property against MCF-7 cells (IC_50_ = 11.91 µM) compared to that of erlotinib (IC_50_ = 4.15 µM). SAR analysis displayed that the conjugation of the hydrazinyl thiazole moiety with 5-nitrofuranyl promoted EGFR suppression efficacy more than other heterocyclic rings such as 5-methylfuranyl, 2-thienyl, 2-pyrrolyl, and 4-pyridinyl. Furthermore, the replacement of 5-nitrofuranyl scaffold with a phenyl moiety substituted by either an EDG or EWG is detrimental to the activity. The docking analysis revealed the establishment of two hydrogen bond acceptors between Lys721 and the nitrogen atoms of the thiazole and benzimidazole rings. Moreover, an arene–cation interaction between Asp831 and the furanyl ring was observed, along with an arene–arene interaction between the thiazole moiety and Phe699 (Figure 18).

**Figure 19:**
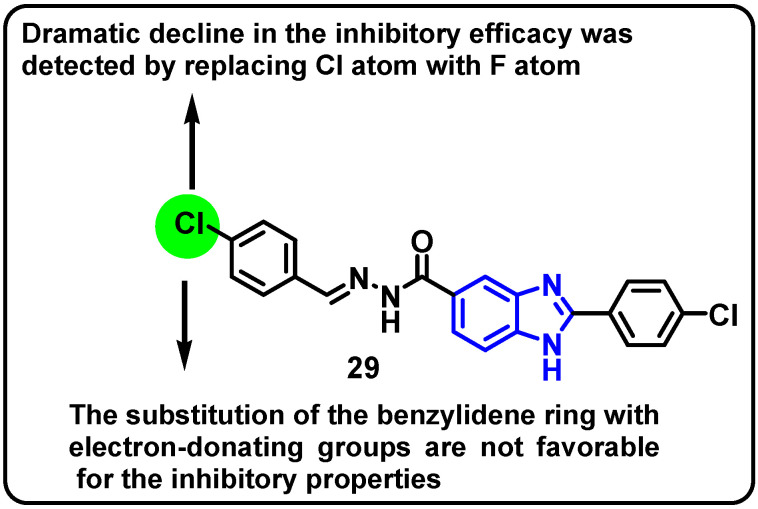
Structure of benzimidazole–hydrazone derivative **29** as EGFR kinase inhibitor.

In 2024, Youssif et al. [67] discovered a novel benzimidazole–hydrazone derivative **29** as a dual inhibitor of EGFR and BRAF^V600E^, exhibiting values of IC_50_ = 0.09 and 0.2 µM, respectively. The SAR investigation demonstrated that the substitution of the benzylidene ring with a chlorine atom was the most potent inhibitor. The exchanging of the Cl atom with a NO_2_ group led to a little reduction in the suppression effect. However, a significant reduction in inhibitory efficacy was seen upon replacing the Cl atom with a F atom. Furthermore, the substitution of the benzylidene ring with an EDG is detrimental to the inhibitory characteristics. Derivative **29** had a significant inhibitory impact against the majority of the evaluated tumor subpanels, with GI_50_ values between 0.97 and 4.93 μM, a selectivity ratio from 0.79 to 1.35, and LC_50_ values ranging from 5.73 to 77.90 μM. Additionally, compound **29** triggered apoptosis by elevating levels of Bax caspase-3 and caspase-8, while diminishing the anti-apoptotic protein Bcl2. The docking study results for EGFR kinase indicated the establishment of a hydrogen bond between the carbonyl group and Met769. A pi-H contact was also observed between the benzimidazole core and Leu 694 (Figure 19).

**Figure 20:**
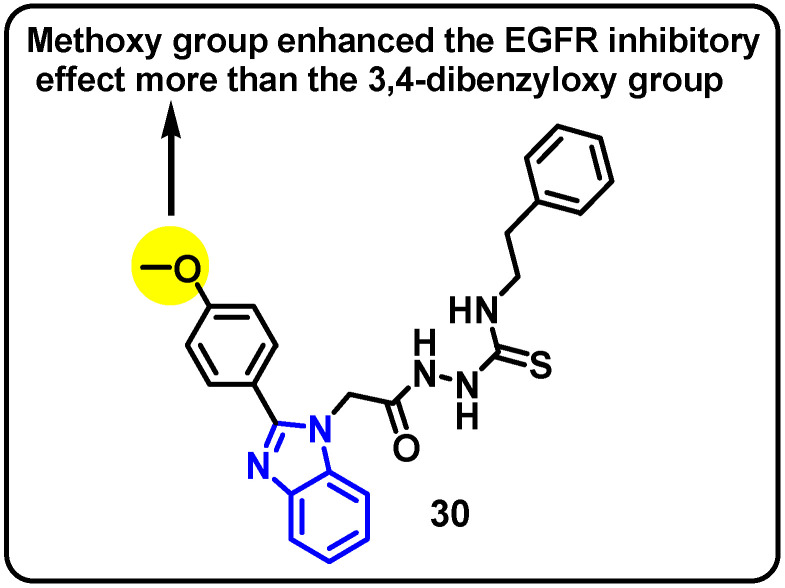
Structure of benzimidazole derivative bearing thiosemicarbazide **30** as EGFR kinase inhibitor.

A new benzimidazole derivative bearing thiosemicarbazide **30** emerged as potent EGFR inhibitor (% inhibition = 68.1%) compared to that of erlotinib (% inhibition = 96.9%) at a concentration of 10 μM. The biological data concluded that the substitution of the phenyl moiety attached to benzimidazole with a methoxy group improved the EGFR inhibitory effectiveness more than the 3,4-dibenzyloxy group. Docking analysis of derivative **30** in the active site of EGFR revealed the interaction between the carbonyl (C=O) group and Lys721 through one H-bond, alongside another two H-bonds between two NH groups and Asp831 were disclosed in addition to hydrophobic interactions with Met769, Phe699, Leu820, Ala719, Met742, Val702, and Ile720 (Figure 20) [68].

**Scheme 3:**
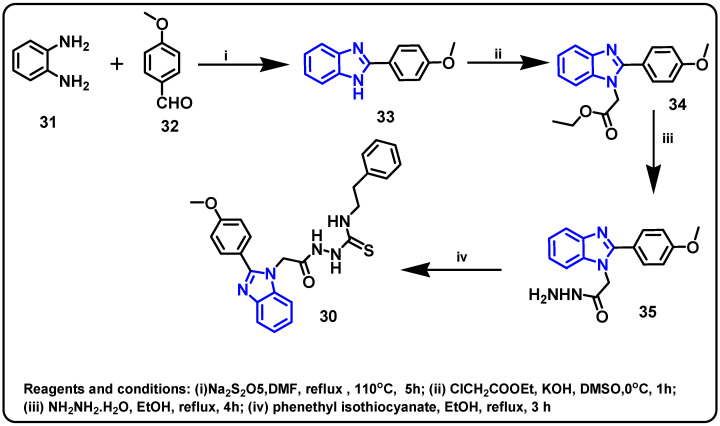
The synthesis of benzimidazole derivative bearing thiosemicarbazide **30**.

The synthesis of a new benzimidazole derivative bearing thiosemicarbazide **30** was accomplished, as illustrated in Scheme 3. The reaction started through the generation of 2-(4-methoxyphenyl)-1H-benzimidazole derivative **33** from the condensation and cyclization of *o*-phenylene diamine **31** with aromatic aldehyde **32**. The later **33** reacted with ethyl chloroacetate to afford compound **34**. The ester group located on derivative **34** converted to a hydrazide group in derivative **35** via reaction with hydrazine hydrate. Conversion of **35** into the target molecule **30** was achieved through reaction with phenethyl isothiocyanate (Scheme 3).

**Figure 21:**
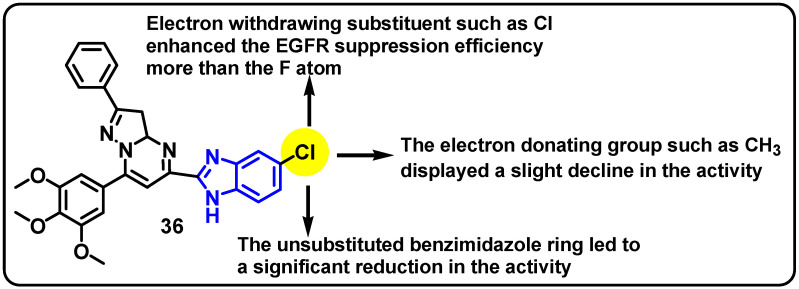
Structure of benzimidazole conjugated with pyrazolo[1,5-*a*]pyrimidine **36** as EGFR kinase inhibitor.

In 2023, a new set of benzimidazole conjugated with pyrazolo[1,5-*a*]pyrimidine was generated and screened for their activity as EGFR inhibitors. Derivative **36** was the most potent EGFR inhibitor (IC_50_ = 0.29 µM), superior to that of erlotinib (IC_50_ = 0.45 µM). Additionally, derivative **36** disclosed significant cytotoxic effectiveness toward HeLa, A549, and MCF-7, with IC_50_ values 9.3, 4.9, and 4.1 µM, respectively, exceeding roscovitine as a reference standard with IC_50_ values 16.1, 27.4, and 17.1 µM, respectively. Interestingly, compound **36** revealed a weak cytotoxic effect against normal lung fibroblasts MRC5 cells (IC_50_ = 47.4 µM). The flow cytometric study demonstrated the accumulation of cells of MCF-7 at the sub-G1 phase upon treatment with compound **36** by 73% compared to erlotinib, as it revealed a 9.3% sub-G1 population. A significant elevation of the levels of apoptotic proteins such as p21, p53, and Bax, as well as a remarkable decline in the level of antiapoptotic protein Bcl-2, were detected by compound **36** in the Western blot analysis. The study of SAR demonstrated that electron-withdrawing substituents such as Cl on the C-5 benzimidazole moiety improved the EGFR suppression efficiency more than the F atom, while the presence of the electron-donating group, such as CH_3,_ displayed a slight decline in the activity. The activity, on the other hand, was significantly diminished as a result of the presence of the unsubstituted benzimidazole ring (Figure 21) [69].

**Scheme 4:**
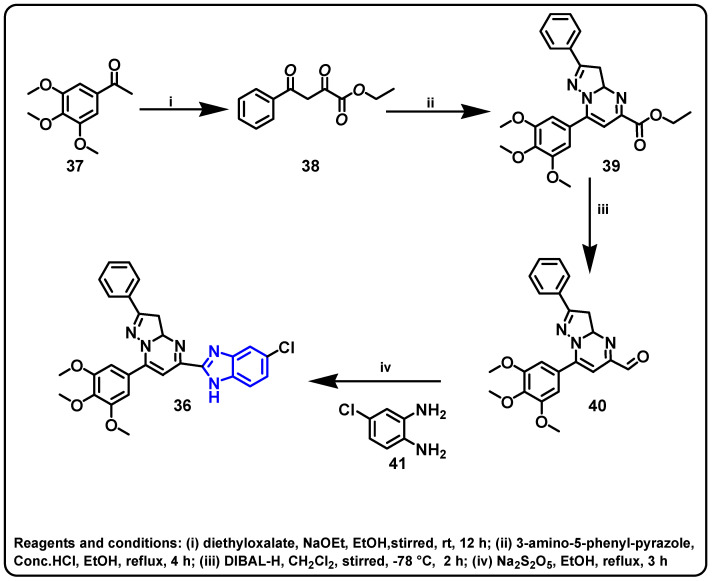
The pathway of synthesis of benzimidazole-pyrazolo[1,5-*a*]pyrimidine hybrid **36**.

It is important to illustrate the pathway to synthesis the benzimidazole conjugated with pyrazolo[1,5-*a*]pyrimidine **36** as depicted in (Scheme 4). The reaction started with isolation of 1,3-diketone **38** from the reaction of 3,4,5-trimethoxy acetophenone **37** with sodium ethoxide and diethyl oxalate in ethanol. Diketone **38** was then cyclized with 3-amino-5-phenyl-1*H*-pyrazole in ethanol to obtain pyrazolo[1,5-*a*]pyrimidine ester derivative **39**. The former derivative **39** underwent reduction reaction to afford pyrazolo[1,5-*a*]pyrimidine-5-carbaldehyde **40**, which subsequently reacted with 4-chlorobenzene-1,2-diamine **41** to furnish benzimidazole-pyrazolo[1,5-*a*]pyrimidine hybrid **36** (Scheme 4).

**Figure 22:**
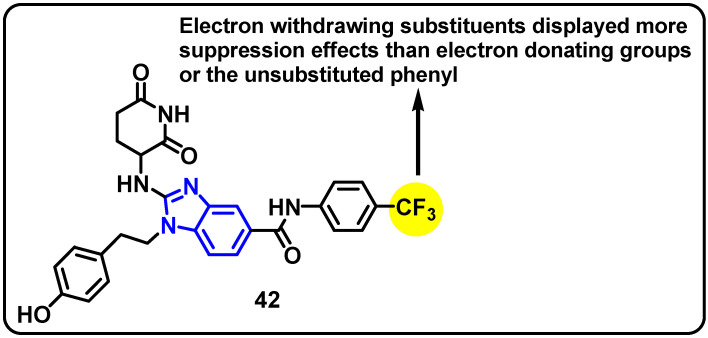
Structure of benzimidazole derivative **42** as EGFR kinase inhibitor.

Theodore and coworkers [70] documented the synthesis and examination of a novel benzimidazole-based derivative targeting EGFR kinase. Derivative **42** presented remarkable suppression efficacy toward both mutant EGFR L858R/T790M and EGFR wild type (WT) (IC_50_ = 5.69 and 4.38 nM, respectively), in contrast to Erlotinib (IC_50_ = 75.64 and 13.55 nM, respectively). It is noteworthy that derivatives with an EWG on phenyl rings linked to the amide moiety exhibited greater suppression effects than those with an EDG and the unsubstituted phenyl. Moreover, derivative **42** exerted considerable cytotoxic effectiveness against HCT116 and MCF-7 cells, with IC_50_ equal to 6.72 and 2.07 µM, respectively. Interestingly, it demonstrated a safety profile toward the non-tumoral Vero cell line (IC_50_ = 56.19 µM). Derivative **42** exhibited the highest docking score of −9.43 kcal/mol, attributed to H-bond interactions with Gly901, Val897, and Lys823 in the EGFR LR/TM active site. In addition to hydrogen bonds, it also established one pi–pi interaction with Tyr-900 and four π–alkyl interactions with Ala822, Val802, Val843, and Ile821 amino acids (Figure 22).

**Figure 23:**
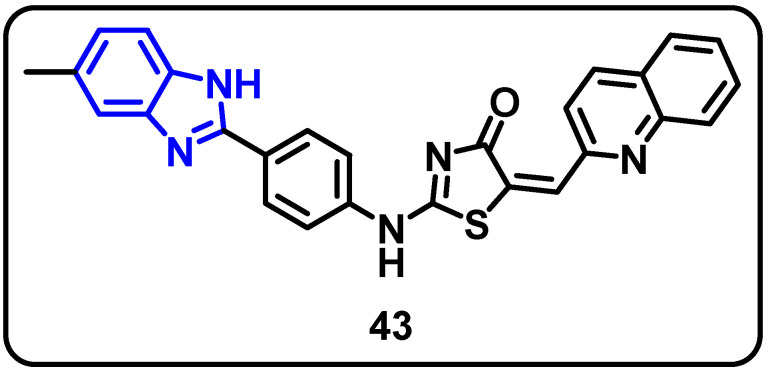
Structure of novel benzimidazole–aminothiazolidinone–quinoline hybrid **43** as EGFR kinase inhibitor.

Furthermore, a novel benzimidazole–aminothiazolidinone–quinoline hybrid **43** emerged as a prominent anticancer agent and EGFR inhibitor. Derivative **43** demonstrated equipotent EGFR inhibitory activity, with IC_50_ = 0.47 μM equal to that of Erlotinib (IC_50_ = 0.44 μM). Furthermore, derivative **43** presented notable cytotoxic effects toward both cell lines of MDA-MB-231 and MCF-7 (IC_50_ = 7.53 and 4.13 μM, respectively), comparable to those of Erlotinib (IC_50_ = 7.18 and 4.32 μM, respectively). Interestingly, it revealed a significant safety profile toward the normal breast epithelial cell line (MCF-10A), with an IC_50_ value of 18.73 μM (Figure 23). Derivative **43** exhibited ADME and toxicity profiles that adhered to Lipinski’s rule of five, indicating advantageous oral drug-like characteristics. Derivative **43** exhibited a score of bioavailability equal to 0.55, comparable to Erlotinib, signifying satisfactory oral absorption efficacy. The anticipated intestinal absorption value was 82.0%, which is near to that of Erlotinib at 97.4%. Moreover, derivative **43** and Erlotinib had advantageous Caco-2 permeability, indicating effective intestinal transport. Furthermore, derivative **43** demonstrated little blood–brain barrier (BBB) permeability (Log BB = −0.902), signifying constrained central nervous system activity. Additionally, derivative **43** was identified as an inhibitor of CYP3A4, a significant enzyme responsible for drug metabolism. Furthermore, inhibition of CYP2C9, CYP2C19, and CYP1A2 was observed, indicating the dangers of drug–drug interactions. Derivative **43** exhibited the highest clearance rate (0.699 mL/min/kg), signifying rapid elimination. The assessed toxicity demonstrated no suppression of hERG I, hence reducing the probability of cardiac arrhythmia. Additionally, it demonstrated hERG II suppression and a potential risk of hepatotoxicity [71].

### 2.2. Vascular Endothelia Growth Factor Receptor (VEGFR) Inhibitors

Angiogenesis, the development of novel blood vessels, is crucial for the proliferation and dissemination of solid tumors [72]. It is widely recognized as essential for sustaining tumor growth beyond a specific threshold, facilitating the delivery of oxygen and essential nutrients. As a result, blocking angiogenesis in tumors has been recognized as a logical strategy for cancer therapy [73]. Since the 1971 proposition that angiogenesis inhibition could serve as an effective cancer treatment strategy, numerous angiogenic regulators have been identified, including platelet-derived growth factor (PDGF), vascular endothelial growth factor (VEGF), angiopoietin, and basic fibroblast growth factor (bFGF). One known member of the kinase class associated with breast cancer development and progression is the vascular endothelial growth factor receptor-2 (VEGFR-2) kinase [74]. VEGFR-2 is markedly overexpressed in numerous solid tumors and is crucial for apoptotic processes. Consequently, blocking VEGFR-2 emerged as a pivotal technique in the pursuit of innovative treatments for malignancies dependent on apoptotic mechanisms [75]. Currently, other VEGFR-2 inhibitors, including sunitinib, lenvatinib, and sorafenib, have received approval for the treatment of diverse cancer types. In addition to the prevalent side effects of sunitinib, including hypertension, tiredness, and diarrhea, individuals also appeared to experience hypothyroidism and cardiotoxic consequences [76,77,78]. Consequently, the identification of more effective and safer VEGFR-2 inhibitors is an urgent necessity.

#### 2.2.1. Imidazole-Based Derivatives as VEGFR-2 Kinase Inhibitors

**Figure 24:**
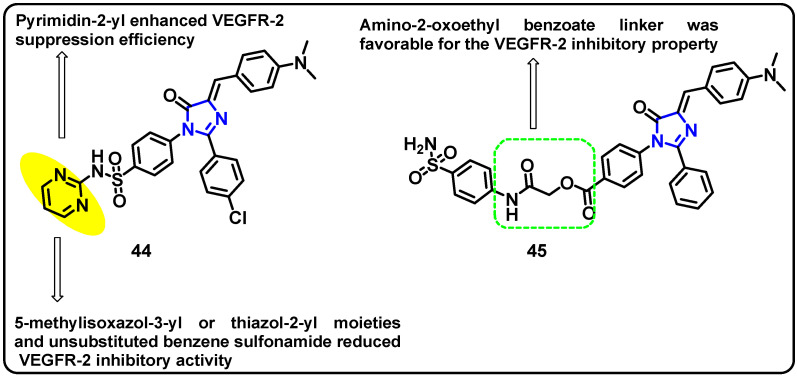
Structures of 1,2,4-trisubstituted imidazolin-5-one derivatives **44** and **45** as VEGFR-2 kinase inhibitors.

Mohamed et al. [79] documented the synthesis and inhibitory evaluation of two novel series of 1,2,4-trisubstituted imidazolin-5-ones targeting VEGFR-2 kinase. The results disclosed that derivatives **44** and **45** presented outstanding VEGFR-2 suppression effects with presented values of IC_50_ = 0.07 and 0.02 µM, respectively, in comparison with the IC_50_ = 0.06 µM of Sorafenib. Furthermore, derivative **44** demonstrated promising anti-proliferative efficiency toward cell lines of MCF-7 and A549 cancer (IC_50_ = 5.86 and 7.66 µM, respectively). In addition, derivative **44** stimulated apoptosis and arrested cell cycle of the MCF7 cell line at the G2−M phase. Moreover, HUVEC analysis results demonstrated that nearly twice the concentration of imidazole derivative **44** compared to Sorafenib is required to exert the desired anti-proliferative efficiency, which indicated its high safety profile (IC_50_ = 178.7 and 89.77 μM, respectively). The docking analysis of compound **44** revealed the formation of a H-bond with Cys-919 in the hinge region, and the imidazolone ring is surrounded with a hydrophobic region containing Phe918, Leu840, and Gly922 amino acids. SAR analysis concluded that the elongation between the imidazolinone and the benzenesulfonamide moiety *via* an amino-2-oxoethyl benzoate linker was favorable for the VEGFR-2 inhibitory property. Moreover, concerning derivative **44**, the substitution of the benzene sulfonamide with pyrimidin-2-yl enhanced VEGFR-2 suppression efficiency, whereas the inhibitory effectiveness was reduced upon the changing of the pyrimidin-2-yl moiety with 5-methylisoxazol-3-yl or thiazol-2-yl moieties and unsubstituted benzene sulfonamide (Figure 24).

**Figure 25:**
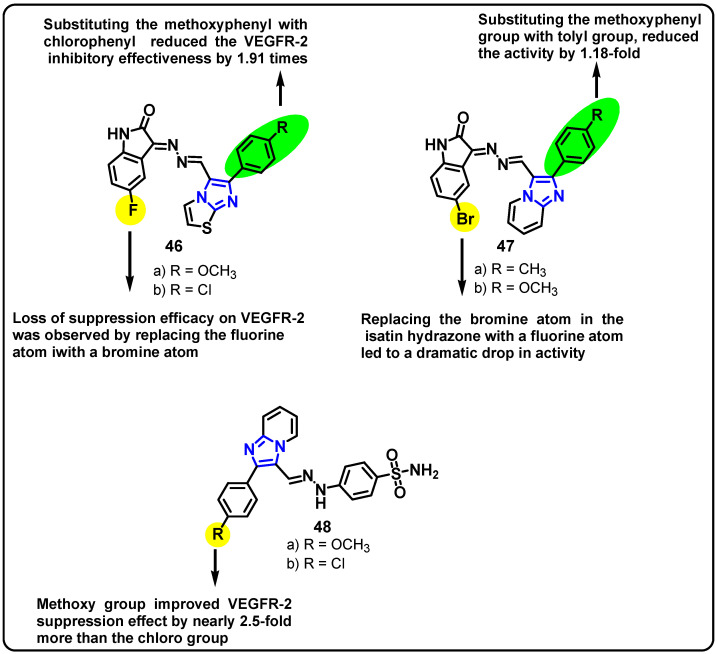
Structures of imidazo[2,1-*b*]thiazoles **46a**,**b** and imidazo[1,2-*a*]pyridines **47a**,**b** and **48a**,**b** as VEGFR-2 kinase inhibitors.

Two sets of imidazo[2,1-*b*]thiazoles and imidazo[1,2-*a*]pyridines coupled with an indolinone moiety were synthesized and evaluated for their capacity to inhibit VEGFR-2 kinase. Among the evaluated candidates, derivatives **46a**,**b** and **47a**,**b** demonstrated exceptional VEGFR-2 inhibitory efficacy with IC_50_ levels of 0.22, 0.42, 0.33, and 0.28 µM, respectively. Regarding the imidazothiazole series with 5-fluoro-isatin-hydrazone revealed that the combination of imidazothiazole and methoxyphenyl (**46a**) had the highest VEGFR-2 inhibitory activity, with an IC_50_ of 0.22 μM, while replacing the methoxyphenyl with chlorophenyl (**46b**) reduced the VEGFR-2 inhibitory effectiveness by 1.91 times. Otherwise, loss of suppression efficacy on VEGFR-2 was observed by replacing the fluorine atom in the isatin-hydrazone with a bromine atom. Moreover, conjugating the imidazopyridines with 5-bromo-isatin-hydrazone disclosed promising outcomes in inhibiting VEGFR-2. Derivative **47b** bearing the methoxyphenyl group was the most efficient VEGFR-2 inhibitor with IC_50_ level 0.28 μM. Exchanging the OCH_3_-phenyl moiety with a tolyl one decreased the activity by 1.18-fold. Furthermore, replacing the Br atom in the isatin-hydrazone with a F atom led to a significant decrease in activity. Furthermore, derivatives **46a** and **47a** displayed selectivity and substantial cytotoxicity against MDA-MB-231 (IC_50_ = 11.90 and 10.88 µM, respectively), comparable to cisplatin (IC_50_ = 11.50 µM). In addition, derivative **47a** exhibited an IC_50_ value of 69.72 μM in normal breast cells (MCF-10A), relative to cisplatin (IC_50_ = 21.75 μM), suggesting a favorable safety profile and selectivity for breast cancer cells. A flow cytometric assay revealed that derivative **47a** stimulated apoptosis in cells of MDA-MB-231 and generated a cell cycle arrest at the G0/G1 phase. Furthermore, derivative **47a** elevated Bax levels by 4.13-fold and diminished Bcl-2 levels by 2.01-fold. The docking analysis of derivative **47a** demonstrated the establishment of two hydrogen bonds between the carbonyl group of the indoline core and the nitrogen atom of the imidazole ring with Cys919. Furthermore, a halogen connection was established between the 5-bromine atom and Arg842. Moreover, derivative **47a** participates in hydrophobic interactions with the amino acids Ala866, Arg1051, Cys1045, Val848, Phe918, Phe1047, Val916, Leu840, Leu1035, Val899, Cys919, Phe921, and Gly922 (Figure 25) [80]. One year later, Elgohary et al. [81] discovered novel imidazo[1,2-*a*]pyridine derivatives **48a** and **48b** as potential VEGFR-2 inhibitors, exhibiting IC_50_ levels 0.576 and 1.453 µM, respectively. The biological findings demonstrated that the substitution of the phenyl moiety connected to the imidazopyridine scaffold with a methoxy group improved the VEGFR-2 suppression effect by approximately 2.5-fold compared to substituting the phenyl ring with a chlorine atom. In addition, derivatives **48a** and **48b** presented the most pronounced cytotoxic effects toward the MDA-MB-231 cell line, with presented values of IC_50_ equal to 4.40 and 4.69 μM, respectively, compared to cisplatin (IC_50_ = 11.50 μM). Furthermore, derivatives **48a** and **48b** stimulate significant G1 phase cell cycle arrest in MDA-MB-231 cells. Both derivatives **48a** and **48b** generated substantial DNA fragmentation and displayed markedly reduced toxicity on MCF-10A normal cells (IC_50_ = 50.87 and 60.64 μM, respectively) in comparison to Cisplatin (IC_50_ = 21.80 μM). The interactions between the powerful derivatives **48a** and **48b** within the active pocket of VEGFR-2 kinase demonstrated the establishment of hydrogen bonds between the oxygen atom of the sulfonamide moieties and Thr926. Moreover, the Cys919 amino acid is engaged in hydrogen bonding with the imidazopyridine core. Furthermore, the 4-OCH_3_-phenyl ring of **48a** engaged with Ala866 via a pi–alkyl contact and with Leu1035 through pi–sigma interaction. Otherwise, derivative **48b**’s para chlorophenyl ring engaged with Leu840 and Ala866 *via* pi–alkyl interactions, and with Leu1035 and Val848 through pi–sigma interactions. Furthermore, the methoxy group engages in pi–alkyl interactions with Val916 and Val848, whereas the chloro group participates in a pi–alkyl interaction with Val916 (Figure 25).

**Figure 26:**
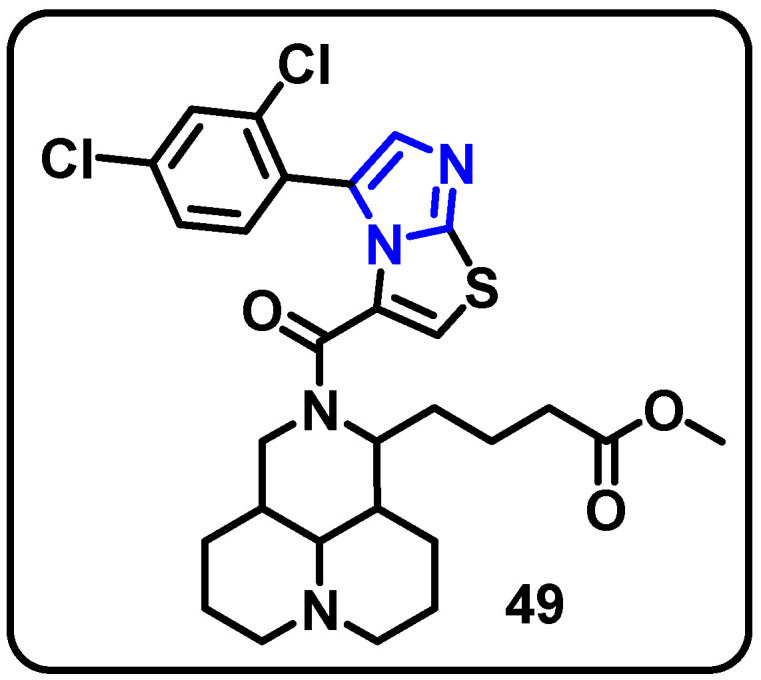
Structure of imidazo[2,1-b]thiazole-matrine hybrid **49** as VEGFR-2 kinase inhibitor.

The production of new imidazo[2,1-*b*]thiazole-matrine hybrids against VEGFR-2 kinase was reported by Zhou et al. [82]. Derivative **49** displayed a significant level of effectiveness in suppressing VEGFR-2, with an IC_50_ level 3.09 μM. In addition, derivative **49** had the most substantial cytotoxic effects toward A549, HCT-116, HGC-27, and Hela cells, with values of IC_50_ = 16.06, 9.52, 9.26, and 13.99 μM, respectively. These values are equivalent to those of sorafenib. It demonstrated weak cytotoxicity against normal gastric mucosal epithelial cells GES-1 with 63.52 μM for the IC_50_. Furthermore, derivative **49** dose-dependently arrested HGC-27b cells in the G0/G1 stage and triggered early and late apoptosis in HGC-27 cells. Derivative **49** attached tightly inside the binding pocket of VEGFR-2. This attachment was achieved through hydrogen bond demonstrated between the nitrogen atom of the imidazo[2,1-*b*]thiazole scaffold and Ala 881. Additionally, two hydrogen bonds were detected between the two carbonyl (C=O) groups with Arg1027 and Leu1049. A pi–anion interaction was identified between the imidazole moiety and Asp814 (Figure 26).

#### 2.2.2. Benzimidazole-Based Derivatives as VEGFR-2 Kinase Inhibitors

**Figure 27:**
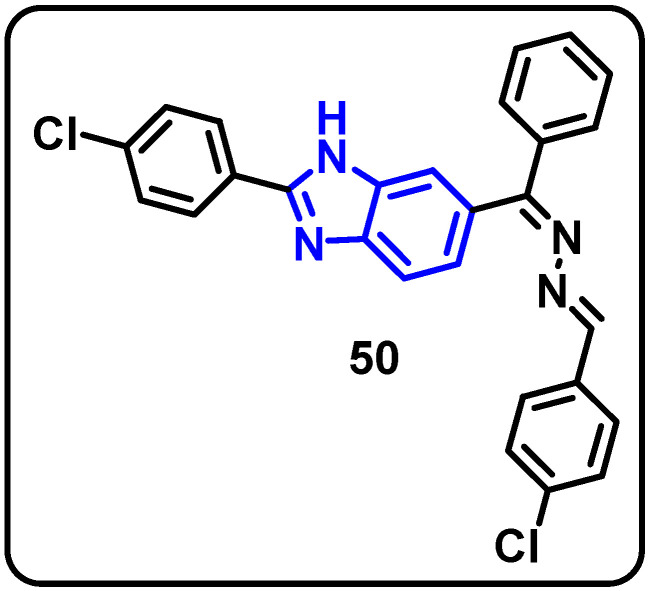
Structure of benzimidazole–Schiff hybrid **50** as VEGFR-2 kinase inhibitor.

A new benzimidazole–Schiff hybrid **50** emerged as remarkable VEGFR-2 inhibitor with % inhibition = 89.89% exceeding that of Sorafenib (% inhibition = 88.17%). Derivative **50** demonstrated outstanding cytotoxic effect against lung cancer NCI-H460 and A549 cells (IC_50_ = 0.85 and 1.88 μM, respectively) in comparison to Sorafenib (IC_50_ = 3.49 and 4.84 μM, respectively). Furthermore, derivative **50** ceased the NCI-H460 cell cycle at both the G1 and S phases. Furthermore, derivative **50** stimulated apoptosis and led to a significant elevation in caspase-9 levels relative to untreated NCI-H460 cells. The benzimidazole–Schiff hybrid **50** exhibited a strong binding affinity within the VEGFR-2 active site (−8.59 Kcal/mol) compared to Sorafenib (−8.74 Kcal/mol). The phenyl ring associated with the hydrazone moiety engages in hydrophobic interactions with Phe1047. Additionally, the hydrazone moiety participates in hydrogen donor and acceptor interactions with the amino acids Glu 885 and Asp 1046, respectively (Figure 27) [83].

**Scheme 5:**
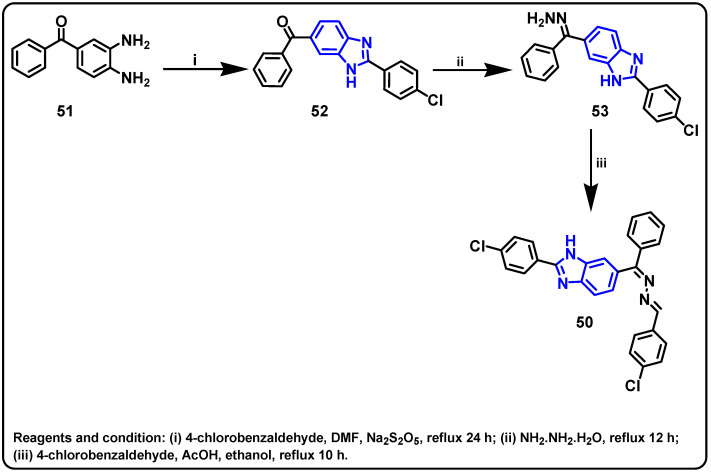
The synthesis of benzimidazole–Schiff hybrid **50**.

The synthesis of a new benzimidazole–Schiff hybrid **50** is achieved as depicted in Scheme 5. *O*-phenylene diamine derivative **51** was converted to benzimidazole derivative **52** via reaction with 4-chlorobenzaldehyde followed by condensation with hydrazine hydrate to afford derivative **53**. The previous derivative **53** was reacted with another molecule of chlorobenzaldehyde to generate the target molecule **50** (Scheme 5).

**Figure 28:**
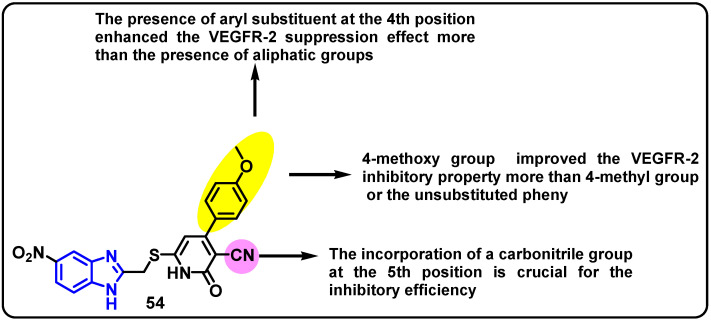
Structure of 5-nitrobenzimidazole–pyrimidine hybrid **54** as VEGFR-2 kinase inhibitor.

In 2024, a new 5-nitrobenzimidazole–pyrimidine hybrid **54** was discovered as a potent VEGFR-2 inhibitor (IC_50_ = 2.83 μM) and a good cytotoxic candidate against HepG2 cells (IC_50_ = 4.37 μM). The biological results demonstrated that the existence of aryl substituent at the fourth position of the pyrimidine ring enhanced the VEGFR-2 suppression effect more than the presence of aliphatic groups. Furthermore, the incorporation of a carbonitrile moiety at the 5th position of the pyrimidine core is crucial for the inhibitory efficiency. Additionally, the introduction of 4-methoxy group on the phenyl connected to the pyrimidine core improved the VEGFR-2 inhibitory property more than 4-methyl group or the unsubstituted phenyl. Derivative **54** displayed a binding energy score of −14.94 kcal/mol in comparison to sorafenib’s score of −15.19 kcal/mol. The C=N and NH groups of the benzimidazole scaffold engage in H-bonding interactions with Cys1045 and Glu885, respectively. The methylenethio spacer engaged with Asp1046. The thiopyrimidine moiety engages in hydrophobic interactions with the amino acids Cys919, Leu840, Phe1047, Val899, Leu1015, Val848, Phe918, and Leu1035. Furthermore, a hydrogen link was identified between the methoxy group and Cys919. The benzimidazole ring engages in hydrophobic interactions with the amino acids Ile892, Ile1044, Leu889, Val899, Val898, Ile888, and Leu1019 (Figure 28) [84].

**Figure 29:**
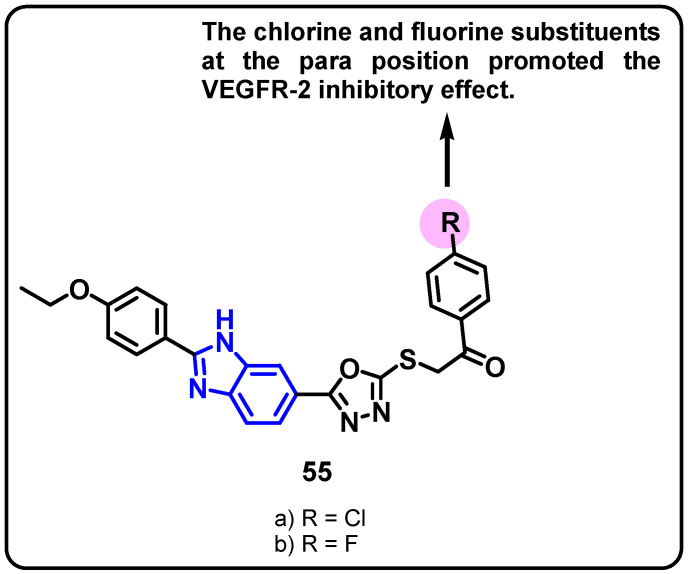
Structures of benzimidazole–oxadiazole hybrids **55a**,**b** as VEGFR-2 kinase inhibitors.

Çevik et al. [85] reported the synthesis of novel benzimidazole–oxadiazole hybrids as potent VEGFR-2 suppressors. Among the examined hybrids, derivatives **55a** and **55b** presented remarkable VEGFR-2 suppression effectiveness with IC_50_ equal to 0.475 and 0.618 µM, respectively. It can be seen from the SAR study that the chlorine and fluorine substituents at the 4th position of the phenyl moiety linked to the oxadiazole ring promoted the VEGFR-2 inhibitory effect. In addition, derivatives **55a** and **55b** were detected to be the most efficient candidates toward MCF-7, A549, and PANC-1 cells, with values of IC_50_ ranging from 0.278 to 5.079 µM. Interestingly, derivatives **55a** and **55b** demonstrated prominent safety profiles on the normal hTERT-HPNE cells with IC_50_ equal to 41.251 and 68.963 µM, respectively. Derivatives **55a** and **55b** revealed strong affinity to VEGFR-2, with energy scores of −10.727 and −10.519 kcal/mol, respectively (Figure 29).

**Figure 30:**
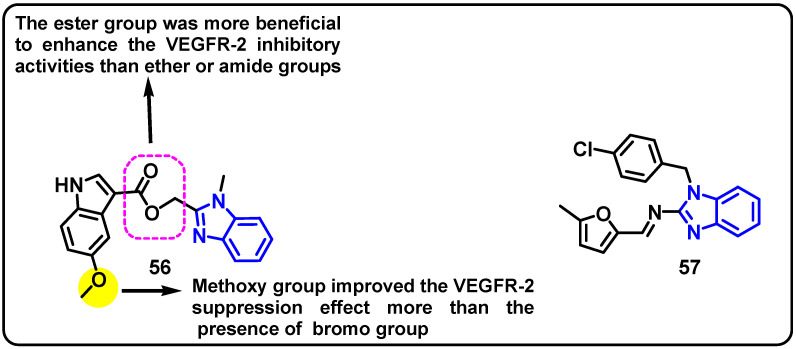
Structures of benzimidazole–indole hybrid **56** and 2-aminobenzimidazole-based derivative **57** as VEGFR-2 kinase inhibitors.

Novel benzimidazole–indole hybrids, interconnected by various linkers, are developed and produced effective VEGFR-2 inhibitors. Among the evaluated hybrids, derivative **56** displayed the highest inhibitory efficacy toward VEGFR-2, with an inhibition percentage of 36.7%. SAR analysis disclosed that the various linking moieties between the benzimidazole and indole scaffolds have prominent effect on the VEGFR-2 inhibitory properties. The substituent’s nature on the indole core influenced the suppression potency. The ester group was more beneficial to enhance the VEGFR-2 inhibitory activities than ether or amide groups. In addition, the presence of an EDG attached to the indole core such as a methoxy group improved the VEGFR-2 suppression effect more than the presence of an EWG as a bromo group. Moreover, derivative **56** demonstrates significant inhibitory efficacy on the proliferation of HT-29, HeLa, MDA-MB-435, and A549 cells, with IC_50_ = 0.3282, 0.2698, 0.4535, and 0.3231 μM, respectively. The docking results indicated that compound **56** was well-integrated into the active pocket of VEGFR-2, exhibiting an energy score of −8.8 kcal/mol, which is comparable to sunitinib’s energy score of −9.4 kcal/mol (Figure 30) [86]. Additionally, the 2-aminobenzimidazole derivative **57** displayed anti-lymphangiogenic and anti-angiogenic effects by targeting VEGF-A/VEGFR-2 signaling in vascular endothelial cells. Derivative **57** demonstrated pi–cation interactions with the His1026 and Lys868 residues inside the binding pocket of VEGFR-2 (Figure 30) [87].

### 2.3. c-Met Kinase Inhibitors

c-Met derived from the protooncogene MET and serves as the tyrosine kinase receptor for hepatocyte growth factor (HGF) [88]. HGF, referred to a scatter factor, binds to c-Met and initiates the autophosphorylation of several tyrosine residues within the intracellular domain. The pathway of HGF/c-Met is crucial for appropriate embryonic development and organ regeneration. Nonetheless, aberrant HGF/c-Met signaling has been recognized in multiple human tumors, including colorectal, brain, lung, gastric, breast, and neck and head cancers [89]. Inhibiting c-Met kinase impact using small-molecule inhibitors is acknowledged as a potentially useful approach for cancer treatment. Thus, c-Met kinase has become a promising target in molecularly targeted cancer therapy [90,91]. So far, several ATP-competitive small molecule c-Met kinase inhibitors have been investigated and developed to inhibit the aberrant c-Met signaling pathway for the treatment of various cancer types [92]. According to the structural characteristics of molecules and their bonding interactions with the c-Met kinase domain, small molecule c-Met kinase inhibitors can be primarily classified as Type I inhibitors and Type II inhibitors [93,94]. Recent data suggest that mutations in specific amino acid residues adjacent to the c-Met kinase active site may confer pharmacological resistance to type I inhibitors. In contrast, it was proposed that type II inhibitors have greater potency against these mutations due to their binding contacts extending beyond the entrance of the c-Met kinase active site [95,96]. Examples of inhibitors for type II c-Met kinase include cabozantinib, foretinib, and altiratinib; nonetheless, they exhibit frequent adverse effects.

Consequently, the invention of more secure and promising c-Met kinase inhibitors is an imperative request.

#### Imidazole-Based Derivatives as c-Met Kinase Inhibitor

**Figure 31:**
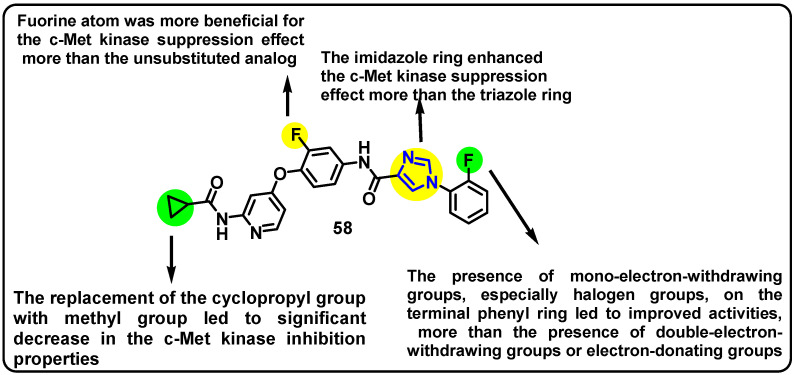
Structure of imidazole-4-carboxamide derivative **58** as c-Met kinase inhibitor.

Liu and his coworkers [97] discovered a new 4-phenoxypyridine derivative bearing imidazole-4-carboxamide **58** as a prominent c-Met kinase inhibitor (IC_50_ = 0.012 μM). The SAR research revealed that derivatives containing the imidazole ring exhibited a greater inhibitory effect on c-Met kinase than those bearing the triazole ring. Furthermore, exchanging the cyclopropyl group with a methyl group led to a substantial reduction in c-Met kinase inhibitory characteristics. The substitution of the phenoxy ring with a fluorine atom was more advantageous for the suppression of c-Met kinase compared to the unsubstituted counterpart. The inclusion of mono-EWG, particularly halogen groups, on the terminal phenyl ring resulted in enhanced activities, surpassing those associated with a double-EWG or EDG. Additionally, derivative **58** exhibited notable anti-proliferative activities against H460, A549, and MKN-45 cell lines, with IC_50_ values of 2.68, 1.92, and 0.64 μM, respectively. Derivative **58**’s binding mode with c-Met kinase revealed the involvement of the NH of cyclopropane carboxamide and the nitrogen atom of pyridine in two hydrogen bonds with Met1160. The imidazole-4-carboxamide moiety forms a hydrogen bond with Asp1222. Furthermore, the 4-F atom on the phenyl group established a halogen–hydrogen bond contact with His1202. The terminal phenyl ring and the pyridyl ring established two pi–pi connections with His1202 and Tyr1159, respectively (Figure 31).

### 2.4. Fibroblast Growth Factor Receptors (FGFR) Inhibitors

The fibroblast growth factor receptor (FGFR) family has become a significant therapeutic target, particularly in oncological conditions over the past decade. It comprises four members (FGFR1 to FGFR4) that are physically analogous receptor tyrosine kinases. Intracellular signaling pathways are initiated when fibroblast growth factor (FGF) ligands bind to fibroblast growth factor receptors (FGFRs). The FGF-FGFR axis is integral to essential biological processes, including proliferation, migration, and differentiation [98,99,100,101,102,103]. Notwithstanding progress in clinical diagnoses and treatment, the prognosis for advanced cancer remains exceedingly poor [104]. Consequently, the inhibition of atypical FGF/FGFR signaling is considered a promising therapeutic strategy for managing cancer therapies. Significant efforts have been made to create small molecule inhibitors that target FGF/FGFR signaling in carcinogenesis. Although numerous agents are under preclinical and clinical trials for various FGFR-related cancers, several have received approval for clinical application. The initial non-selective generation of FGFR inhibitors that bind to the conserved ATP-binding region in receptor tyrosine kinases includes Dovitinib [105], Nintedanib [106], and Cediranib [107]. They have been employed to address multiple types of solid tumors, such as breast malignancies, squamous cell lung cancers, and metastatic urothelial cancers [108,109]; nevertheless, their application is associated with some undesirable side effects [110,111,112]. To address these shortcomings, researchers have developed a second generation of extremely effective and selective FGFR TKIs, such as Infigratinib [113] and Erdafitinib [114]. However, these selective FGFR inhibitors display numerous adverse effects, like hyperphosphatemia, diarrhea, dry skin, and mucosal disturbance [115,116,117]. Thus, the finding of effective, safe, and selective FGFR inhibitors is urgently needed.

#### 2.4.1. Imidazole-Based Derivatives as FGFR Kinase Inhibitor

**Figure 32:**
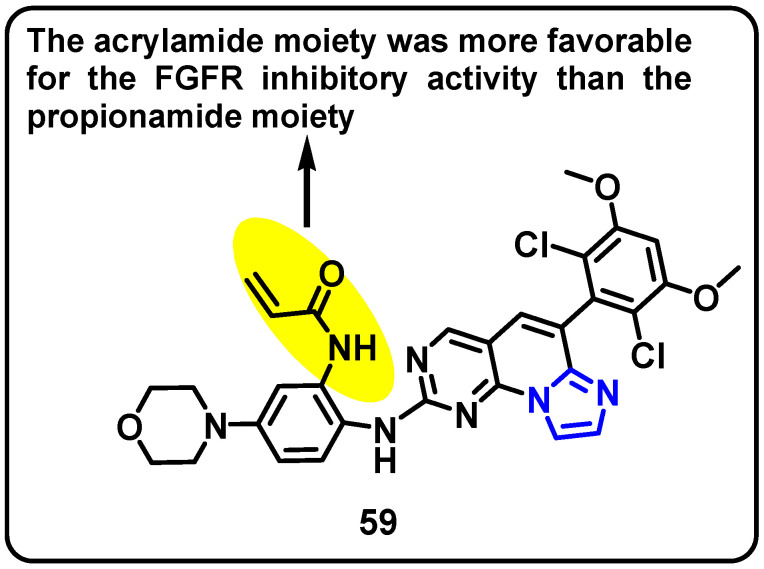
Structure of imidazo[1′,2′:1,6]pyrido[2,3-*d*]pyrimidine derivative **59** as FGFR kinase inhibitor.

Kim and his colleagues [118] documented the synthesis of novel derivatives of imidazo[1′,2′:1,6]pyrido[2,3-*d*]pyrimidine as selective inhibitors of FGFR 1–4. Among the synthesized candidates, derivative **59** emerged as the most significant inhibitor of FGFR1, 2, and 4, with IC_50_ concentrations equal to 8, 4, and 3.8 nM, respectively. The biological data demonstrated that the acrylamide moiety was more beneficial for FGFR inhibitory activity than the propionamide moiety. Moreover, the in vivo efficacy of derivative **59** on the growth of subcutaneous xenografted Huh7 cell tumors in nude mice demonstrated that the oral administration of **59** yielded anticancer activity, achieving a tumor growth inhibition of 82% on day 15, compared to the FDA-approved FGFR inhibitor erdafitinib, which exhibited a maximum tumor growth inhibition of 47.2% on the same day. Moreover, derivative **59** had a significant inhibitory effect on the proliferation of FGF19-overexpressing hepatocellular carcinoma cells, with GI_50_ values ranging from 4.4 to 45 nM. The covalent docking of derivative **59** at the FGFR4 binding site resulted in a stable 42-adducted FGFR4, produced via a covalent bond between acrylamide and the hinge Cys552 residue. In addition, derivative **59** formed multiple H-bonds with Arg483, Lys471, and Ala553 (Figure 32).

#### 2.4.2. Benzimidazole-Based Derivatives as FGFR Kinase Inhibitor

**Figure 33:**
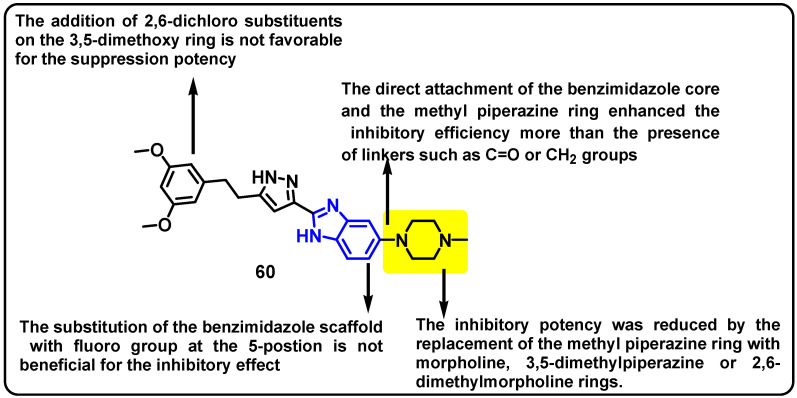
Structure of benzimidazole–pyrazole hybrid **60** as FGFR kinase inhibitor.

Yamani and his coworkers [119] synthesized new benzimidazole–pyrazole hybrids and investigated their suppression effectiveness against FGFR (1-3). Among the screened hybrids, derivative **60** (CPL304110) was recognized as a selective and powerful pan-FGFR inhibitor for FGFR1, -2, and -3, exhibiting IC_50_ equal to 0.75, 0.50, and 3.05 nM, respectively. SAR study disclosed that the conjugation of the benzimidazole scaffold with methyl piperazine ring demonstrated the most effective inhibitory properties against FGFR (1-3). Otherwise, the inhibitory potency diminished when replacing the methyl piperazine moiety with morpholine, 3,5-dimethylpiperazine, or 2,6-dimethylmorpholine rings. Furthermore, the substitution of the benzimidazole scaffold with a fluoro group at the 5th position does not enhance the inhibitory action. Additionally, the direct attachment of the benzimidazole core and the methyl piperazine ring enhanced the inhibitory efficiency more than the presence of linkers such as C=O or CH_2_ groups. The incorporation of 2,6-dichloro substituents on the 3,5-dimethoxy ring is detrimental to the suppression potency. Derivative **60** exhibited an in vitro anti-proliferative effect against seven cell lines with FGFR abnormalities, demonstrating IC_50_ values ranging from 79.08 to 392.80 nM, indicating a significant suppression of growth in FGFR-dependent cancer cell lines compared to those without FGFR gene abnormalities. Additionally, derivative **60** is presently being assessed in a phase I clinical trial for the treatment of bladder, stomach, and squamous cell lung malignancies (01FGFR2018; NCT04149691). The docking investigation of derivative **60** with FGFR1 revealed hydrogen bond interactions between the NH of the benzimidazole and the N of the pyrazole fragments with Ala564. Furthermore, the nitrogen atom of the pyrazole established a hydrogen bond with Glu562, whereas the oxygen atom of one methoxy group produced a hydrogen bond with Asp641 (Figure 33).

### 2.5. FMS-like Tyrosine Kinase 3 (FLT3) Inhibitors

FMS-like tyrosine kinase 3 (FLT3) is a receptor tyrosine kinase belonging to the class III PDGF receptor family [120]. It comprises an external immunoglobulin-like domain for FLT3 ligand interaction, a juxtamembrane (JM) region, and a cytoplasmic kinase domain that includes a kinase insert domain [121]. Ligand binding induces FLT3 dimerization, resulting in autophosphorylation of the kinase domain, which then activates downstream pathways including the RAF-MEK pathway, JAK-STAT pathway, and PI3K-AKT pathway [120,121]. Mutations that activate FLT3, including internal tandem duplications (ITDs) and point mutations in the tyrosine kinase domain, are frequently observed in acute myeloid leukemia (AML) cases. The ITD mutation, characterized by the duplication of an exon inside the JM domain, impairs the domain’s autoinhibitory activity, resulting in its constitutive activation [122]. Approximately 25% of AML patients have this characteristic [123]. Furthermore, the ITD mutation has been identified as a causal change and an appropriate therapeutic target for AML [124]. Targeting both FLT3-Wild Type and mutant FLT3 is a viable method for the treatment of Acute Myeloid Leukemia (AML) due to the significance of FLT3 in this condition [125].

All recognized FLT3 inhibitors can be classified as type I or type II according to their binding mechanism. Type I inhibitors are competitive inhibitors that bind to the active conformation of FLT3 (DFG-in form). Sunitinib, midostaurin, lestaurtinib, crenolanib, and gilteritinib are classified as type I FLT3 inhibitors that exhibit a strong affinity for its active conformation. Type I inhibitors, however, lack selectivity for FLT3 and demonstrate a robust binding affinity for several kinases because of the considerable similarities in their ATP binding sites. Type II inhibitors interact with the DFG-out conformation, the inactive state, and also bind to an adjacent hydrophobic region near the ATP-binding pocket [126,127]. Type II inhibitors generally demonstrate enhanced selectivity for particular kinases owing to the comparatively less conserved characteristics of hydrophobic pockets relative to ATP binding sites. Sorafenib and quizartinib are classified as type II FLT3 inhibitors. Among the FLT3 inhibitors mentioned, only two agents, midostaurin (RydaptVR) and gilteritinib (XospataVR), obtained FDA clearance for the treatment of FLT3-mutated AML in 2017 and 2018, respectively. In 2019 [128], Quizartinib received regulatory approval in Japan for patients with FLT3-ITD positivity; nonetheless, it led to dose-dependent toxicity [129]. Consequently, the discovery of novel, effective FLT3 inhibitors with minimal adverse effects remains a pressing necessity.

#### 2.5.1. Imidazole-Based Derivatives as FLT3 Kinase Inhibitors

**Figure 34:**
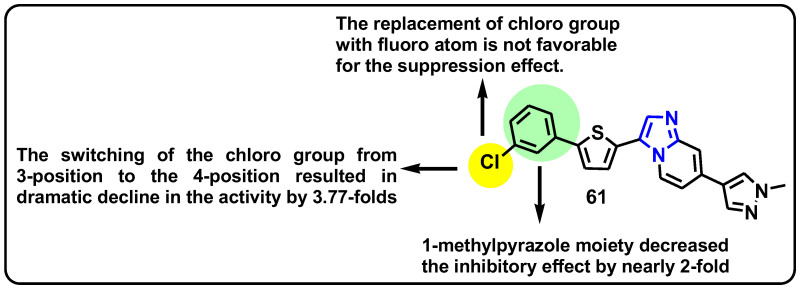
Structure of imidazo[1,2-a]pyridine-thiophene derivative **61** as FLT3 kinase inhibitor.

Fms-like tyrosine kinase 3 (FLT3) has been established as a therapeutic target for acute myeloid leukemia (AML). Zhang et al. [130] discovered an imidazo[1,2-*a*]pyridine-thiophene derivative **61** as a type-I inhibitor of FLT3, with an IC_50_ = 0.053 µM. SAR analysis revealed that the conjugation of the thiophene core with the 3-chlorophenyl ring exhibited the most significant FLT3 inhibition efficacy. The incorporation of the thiophene ring with the 1-methylpyrazole moiety reduced the inhibitory action by approximately two-fold. The relocation of the chloro group from the 3-position to the 4-position led to a significant reduction in activity by 3.77-fold. Moreover, exchanging the chloro atom with a fluoro atom is not advantageous for the suppressing effect. Moreover, the coupling of the thiophene core with the 2,6-dimethoxyphenyl ring did not enhance the inhibitory effect. Moreover, derivative **61** exhibited notable anti-proliferative effects against FLT3-ITD-, FLT3-ITD^F691L^-, and FLT3-ITD ^D835Y^-driven AML cell lines, with IC_50_ levels 0.16, 1.29, and 1.50 µM, respectively (Figure 34).

**Figure 35:**
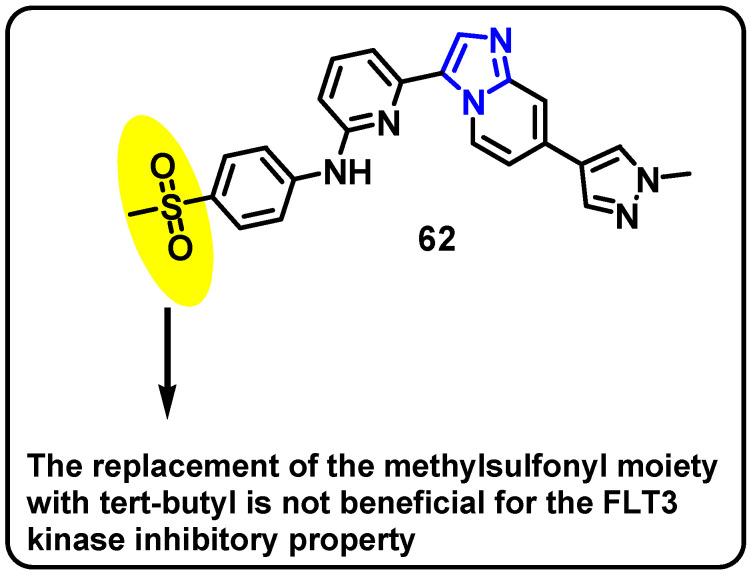
Structure of imidazo[1,2-a]pyridine-pyridine derivative **62** as FLT3 kinase inhibitor.

In 2024, imidazo[1,2-*a*]pyridine-pyridine derivative **62** emerged as a potent FLT3 inhibitor, with an IC_50_ value of 7.94 nM. The biological result illustrated that the replacement of the methylsulfonyl moiety with tert-butyl is not beneficial for the FLT3 kinase inhibitory property. Moreover, the inhibitory effects of derivative **62** on MOLM14-D835Y and MOLM14-F691L cells (IC_50_ = 3.16 and 8.34 nM, respectively) are more balanced than gilteritinib (IC_50_ = 7.83 and 58.78 nM, respectively). Also, there are no anti-proliferative effects observed in either MDA-MB-231 and HEK-293 cells, indicating that derivative **62** is not cytotoxic. The docking investigation revealed that derivative **62** binds to FLT3-WT with its amino-pyridine warhead oriented towards the ATP-binding pocket, establishing hydrogen connections with the Cys695 and Cys694 residues, as well as aromatic-hydrogen interactions with Cys694, Leu616, Phe830, and Cys695 (Figure 35) [131].

#### 2.5.2. Benzimidazole-Based Derivatives as FLT3 Kinase Inhibitor

**Figure 36:**
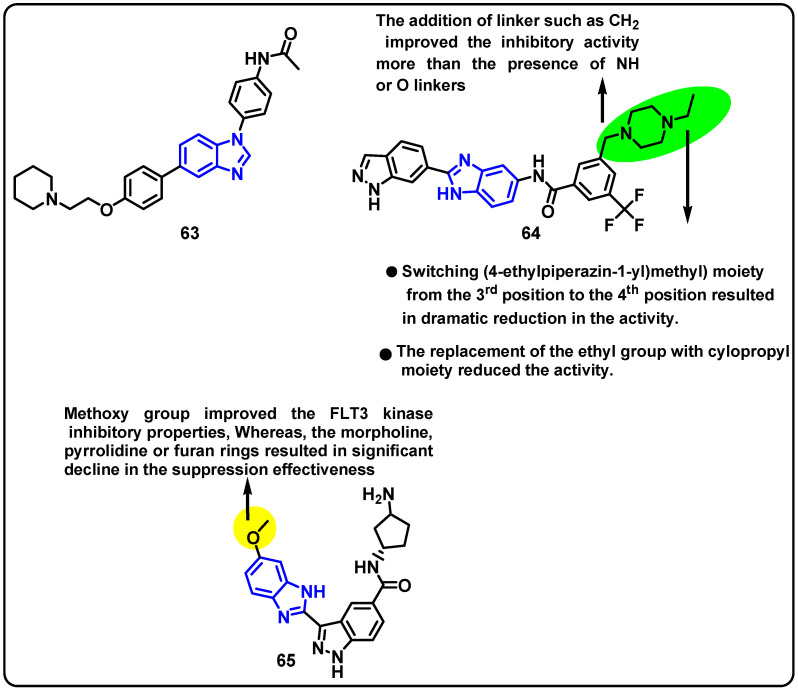
Structures of benzimidazole derivative **63** and benzimidazole–indazole hybrids **64** and **65** as FLT3 kinase inhibitors.

Dokla et al. [132] developed a new benzimidazole derivative **63**, which exhibited inhibitory efficacy against wild-type FLT3, FLT3-Internal tandem duplications (FLT3-ITD), and FLT3-D835Y (FLT3-TKD) mutations, with IC_50_ values of 43.8, 97.2, and 92.5 nM, respectively. Derivative **63** exhibited a good anti-proliferative impact against FLT3-ITD positive acute myeloid leukemia (AML) cell lines, with MV4-11 IC_50_ = 38.8 ± 10.7 nM and MOLM-13 IC_50_ = 54.9 ± 4.1 nM. Furthermore, **63** induced apoptotic and necrotic cell death, as well as G0/G1 cell cycle arrest. Notably, derivative **63** exhibited no cytotoxic effects on either normal murine hepatocyte (BNL) or rat cardiomyoblast (H9c2) cells. Derivative **63** is optimally accommodated into the active site of FLT3, with the nitrogen of the benzimidazole core forming a hydrogen bond with Cys694. The acetamido group established a hydrogen bond with Asp829. Furthermore, the nitrogen of the piperidine molecule established an additional hydrogen bond with the Cys695 amino acid (Figure 36). Benzimidazole–indazole hybrid **64** has been identified as a novel FLT3 inhibitor, demonstrating significant efficacy against FLT3 with an IC_50_ of 41.6 nM, as well as against FLT3-ITD (W51) and FLT3-TKD (D835Y) mutants, with IC_50_ level 22.8 nM and 5.64 nM, respectively. A SAR study demonstrated that the conjugation of benzimidazole with *N*-ethyl piperazine improved inhibitory efficiency, while exchanging the ethyl group with a cyclopropyl moiety diminished inhibitory effectiveness. The incorporation of linkers such as CH_2_ between the phenyl group and the basic amine substituent enhanced the inhibitory activity more than the inclusion of NH or O linkers. Furthermore, relocating the (4-ethylpiperazin-1-yl)methyl moiety from the 3rd position to the 4th position led to a significant decrease in activity. The docking analysis of derivative **64** into the ATP binding site of FLT3 (PDB:4RT7) revealed the establishment of a hydrogen bond between the NH of the indazole and Cys694, as well as an additional hydrogen bond disclosed between the N-H of the amide bond and Asp829. Additionally, the benzimidazole framework established a pi–pi interaction with Phe830 and Phe691. The positively charged nitrogen in the ethyl piperazine ring established a pi–cation interaction with His809 (Figure 36) [133]. In 2023, the novel benzimidazole–indazole hybrid **65** emerged as a promising FLT3 kinase inhibitor. Derivative **65** exhibited exceptional inhibitory effects on FLT3 and FLT3/D835Y, with IC_50_ equal to 0.087 and 0.061 nM, respectively. The SAR investigation showed that substituting the benzimidazole core with a methoxy group enhanced the FLT3 kinase inhibitory characteristics, whereas the substitution of the benzimidazole scaffold with morpholine, pyrrolidine, or furan rings led to a marked decrease in suppression efficacy. Furthermore, derivative **65** exhibited a significant anti-proliferative action against the AML cell line MV4-11, with a GI_50_ of 0.14 nM. Additionally, derivative **65** demonstrated considerable anti-proliferative efficacy in the mutant FLT kinase-expressing Ba/F3 cell lines, specifically F691L (GI_50_ = 7.05 nM) and FLT-D835Y (GI_50_ = 0.66 nM) (Figure 36) [134].

### 2.6. Multi-Targeting Tyrosine Kinase Inhibitors

#### 2.6.1. Imidazole-Based Derivatives as Multi-Targeting Tyrosine Kinase Inhibitors

**Figure 37:**
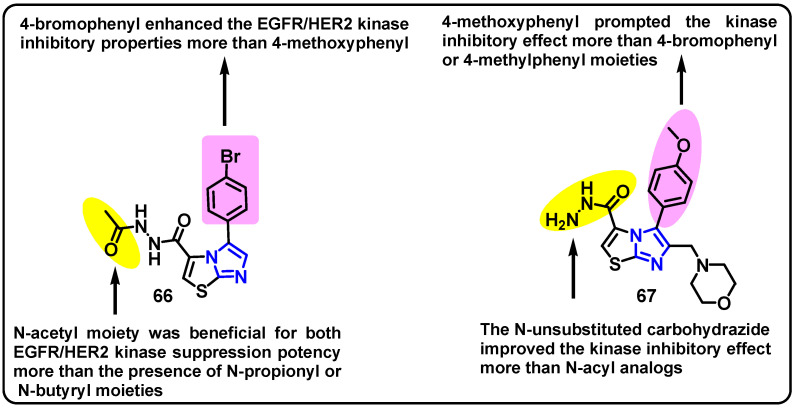
Structures of imidazo[2,1-*b*]thiazole derivatives **66** and **67** as multi-targeting tyrosine kinase inhibitors.

In 2022, two novel imidazothiazole derivatives, **66** and **67**, were identified as dual EGFR/HER2 kinase inhibitors, with IC_50_ = 0.153 and 0.122 µM for EGFR and 0.108 and 0.078 µM for HER2, respectively. The biological data concluded that, concerning 5-(4-substituted-phenyl)-*N*′-acyl-imidazo[2,1-*b*]thiazole-3-carbohydrazide derivatives, the presence of the *N′*-acetyl moiety was beneficial for both EGFR/HER2 kinase suppression potency more effectively than the presence of *N′*-propionyl or *N*′-butyryl moieties. Additionally, the incorporation of the imidazo[2,1-*b*]thiazole core with 4-bromophenyl significantly improved the EGFR/HER2 kinase inhibitory capabilities more than 4-methoxyphenyl. Concerning the 5-(4-substituted phenyl)-6-(morpholino-methyl)imidazo[2,1-*b*]thiazole-3-carbohydrazide derivatives, the *N′*-unsubstituted carbohydrazide exhibited a superior kinase inhibitory activity compared to the *N′*-acyl analogs. The substitution of the imidazo[2,1-*b*]thiazole scaffold with 4-methoxyphenyl enhanced the kinase inhibitory action more than the 4-bromophenyl or 4-methylphenyl groups. Furthermore, derivatives **66** and **67** showed considerable broad-spectrum anti-proliferative efficacy against the evaluated cell lines (HepG2, HCT-116, PC3, MCF-7, and HeLa). Derivative **66** exhibited IC_50_ values of 3.48, 6.08, 12.74, 1.83, and 4.78 μM, whereas derivative **67** demonstrated IC_50_ values of 12.18, 2.37, 16.18, 18.39, and 9.43 μM. Furthermore, derivatives **66** and **67** stimulated their cytotoxicity through cell cycle arrest in the G1/S and G1 phases, respectively, and promoted apoptosis rather than necrosis in the MCF-7 breast cancer cell line. Notably, derivatives **66** and **67** demonstrated a modest cytotoxic effect on the WI-38 cell line, with high IC_50_ values of 54.18 and 36.84 μM, respectively. The in vivo anti-breast cancer assay demonstrated substantial tumor volume decrease in the **66-** and 50-treated groups (76.5% and 65.3%, respectively), accompanied by body weight reductions of 17.4% and 7.4%, respectively. Furthermore, derivatives **66** and **67** elevated the caspase-3 score to 1.33 and 1.17, respectively, which is approximately equivalent to that of doxorubicin (1.5) (Figure 37) [135].

**Figure 38:**
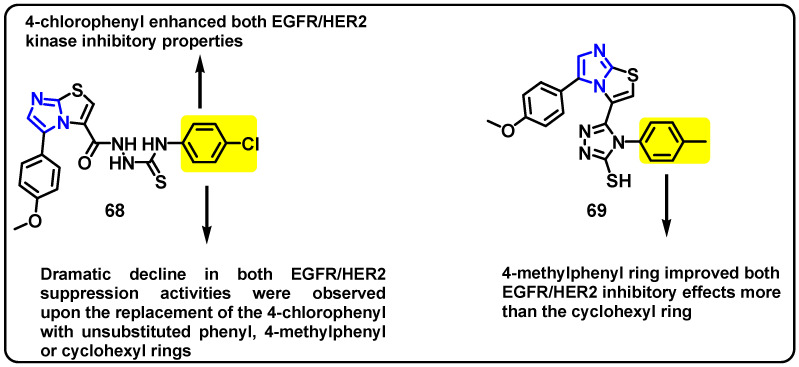
Structures of imidazo[2,1-*b*]thiazole derivatives **68** and **69** as multi-targeting tyrosine kinase inhibitors.

Two years later, two further imidazothiazole analogs, **68** and **69**, were recognized as promising dual EGFR/HER2 kinase inhibitors (EGFR; IC_50_ = 0.087 and 0.099 µM, respectively; HER2; IC_50_ = 0.072 and 0.051 µM, respectively). Concerning the hydrazinecarbothioamide-based derivatives, it was revealed that the connection of the hydrazinecarbothioamide with 4-chlorophenyl enhanced both EGFR/HER2 kinase inhibitory properties. A significant reduction in suppression efficacy was noted following the exchanging of the 4-chlorophenyl group with unsubstituted phenyl, 4-methylphenyl, or cyclohexyl rings. Regarding the triazole-based derivatives, it was concluded that the attachment of the triazole moiety with 4-methylphenyl ring improved both EGFR/HER2 inhibitory effects more than the cyclohexyl ring. In addition, derivatives **68** and **69** demonstrated remarkable broad spectrum anti-proliferative efficiency on the tested cell lines (MCF-7, PC3, HepG2, HCT-116, HEp-2, and Hela). Derivative **68** showed IC_50_ values of 1.81, 14.39, 2.36, 10.67, 9.05, and 5.96 μM, respectively, while derivative **69** revealed IC_50_ values of 4.95, 13.22, 7.28, 9.57, 8.42, and 3.78 μM, respectively. Derivatives **68** and **69** exhibited a modest cytotoxic effect on the WI-38 cell line, with IC_50_ = 17.46 and 35.25 μM, respectively, compared to Sorafenib and Doxorubicin, which had IC_50_ = 10.65 and 6.72 μM, respectively. Cell cycle research revealed that derivative **68** induced cell cycle arrest at the G2/M phase in HepG2 cells and at the G0/G1 phase in MCF-7 cells, indicating that it may exert its cytotoxic effects through distinct mechanisms of action. Conversely, derivative **69** induced cell cycle arrest at the G2/M stage in HeLa cells. The in vivo anticancer efficacy of derivative **68** demonstrated that 20 days post-treatment, the estimated percentage reduction in tumor volume was around 34.6% for derivative **68** and 36.5% for Doxorubicin. This signified a comparably similar percentage of tumor volume reduction in both cohorts. Derivatives **68** and **69** were successfully docked into the active site of the EGFR kinase, yielding docking energy scores of −8.4993 and −7.9215 kcal/mol, respectively. The Lys745 amino acid residue engages in hydrogen bonding with the sulfur atom of hydrazinecarbothioamide in derivative **68** and the thiol group on the triazole moiety in derivative **69**. Lys797, Leu844, Leu788, Leu777, Leu718, Ala743, and Val726 amino acids participate in hydrophobic interactions with both analogs. Conversely, derivatives **68** and **69** exhibited optimal fitting within the HER-2 active site, with docking energy scores of −8.5885 and −7.7808 kcal/mol, respectively. Derivative **68** forms two hydrogen bonds between the NH groups of the hydrazinecarbothioamide moiety with the amino acid residues Thr862 and Asp863. Additionally, hydrophobic interactions were noted between derivative **68** and several amino acid residues of the enzyme, specifically Met801, Leu726, Val734, Met774, Cys805, Ala751, Leu864, Gln799, Leu785, and Leu852. Alternatively, derivative **69** established a sulfur connection between the sulfur atom of the thiazole ring and Met801. Hydrophobic interactions were detected with amino acid residues, including Leu800, Cys805, Ala751, Leu726, Lys753, Leu852, Leu796, and Val734 (Figure 38) [136].

**Scheme 6:**
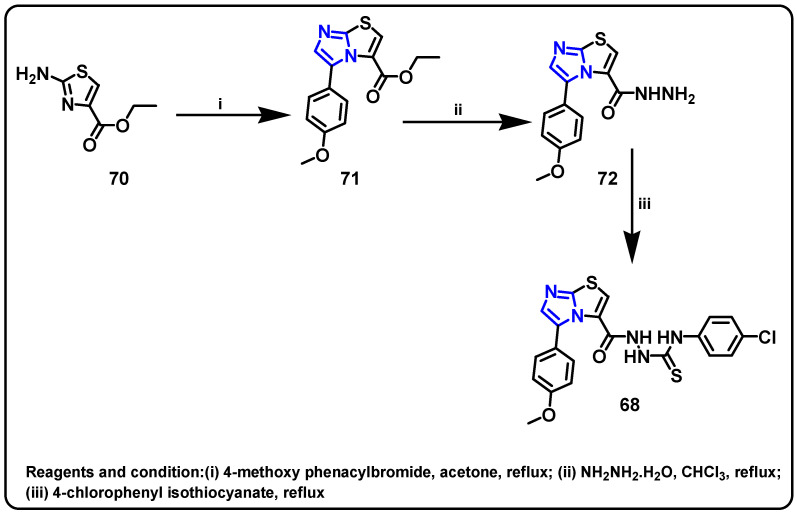
The synthesis of imidazothiazole derivative **68**.

The reaction of ethyl 2-aminothiazole-4-carboxylate **70** with 4-methoxy phenacyl bromide in refluxing acetone afforded the imidazo[2,1-*b*]thiazole-3-carboxylate derivative **71**. The carboxylate derivative **71** was treated with hydrazine hydrate in chloroform to obtain the carbohydrazide analog **72**. The target imidazothiazole analog **68** was prepared via reacting carbohydrazide derivative **72** with 4-chlorophenyl isothiocyanate (Scheme 6).

**Figure 39:**
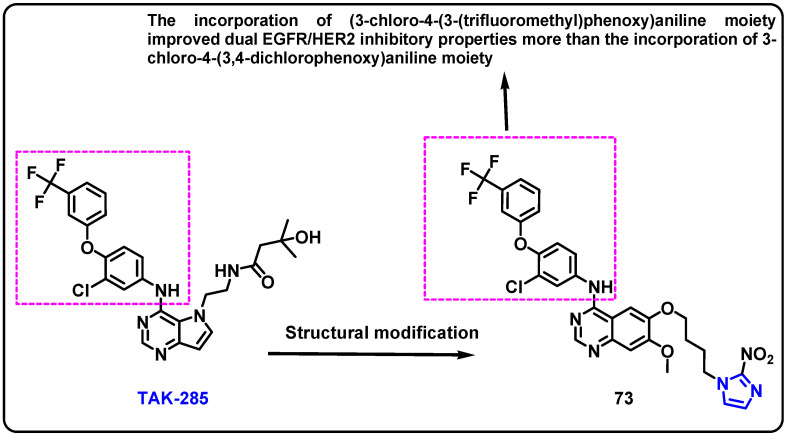
Structures of TAK-285 and TAK-285-imidazole hybrid **73** as multi-targeting tyrosine kinase inhibitors.

Son et al. [137] reported the synthesis of novel TAK-285 compounds and their pharmacological assessment as dual EGFR/HER2 inhibitors. Within the investigated candidates, derivative **73** displayed exceptional dual inhibitory activities against EGFR and HER2, with IC_50_ equal to 2.3 and 234 nM, respectively, compared to Staurosporine (IC_50_ = 88.1 and 35.5 nM, respectively). The biological findings demonstrated that the inclusion of the 3-chloro-4-(3-(trifluoromethyl)phenoxy)aniline moiety enhanced dual EGFR/HER2 inhibitory capabilities more effectively than the 3-chloro-4-(3,4-dichlorophenoxy)aniline moiety. Additionally, derivative **73** exhibited significant cytotoxicity against prostate cancer (PC3), with an IC_50_ equal to 1.0 nM. It also halted the cell cycle of PC3 cells at the G2/M phase. Derivative **73** exhibited a favorable binding score of −8.79 kcal/mol within the EGFR active site, establishing one hydrogen bond with Lys692 and three pi–H interactions with Val702, Arg817, and Cys773. Conversely, derivative **73** exhibited a binding score of −10.64 kcal/mol at the binding region of the HER2 receptor. It established two hydrogen bonds with Cys805 and Met801 (Figure 39).

**Figure 40:**
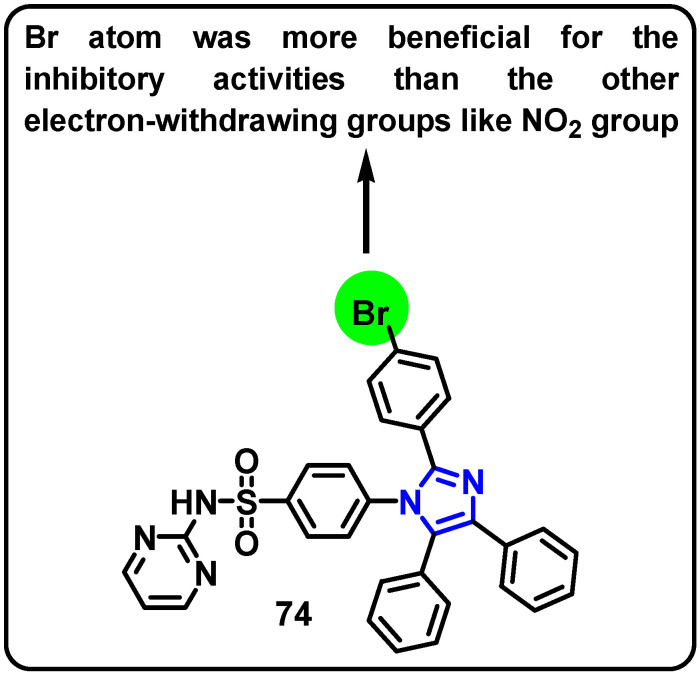
Structure of tetrasubstituted imidazole–pyrimidine–sulfonamide hybrid **74** as multi-targeting tyrosine kinase inhibitor.

In 2023, Alghamdi and coworkers [138] discovered new tetrasubstituted imidazole–pyrimidine–sulfonamide hybrid **74** as a potent dual HER2/EGFR inhibitor. It displayed considerable inhibitory effects against HER2 and two EGFR mutants, L858R and T790M, with values of IC_50_ = 81, 59, and 49 ng/mL, respectively. The substitution of the 2-phenyl group with a 4-Br atom was more beneficial for the inhibitory activities than other EWGs, such as the 4-NO_2_ group. Derivative **74** demonstrated extensive anticancer activity across a panel of 60 cell lines at a single dosage of 10 μM. Furthermore, derivative **74** triggered apoptosis and halted the cell cycle of MCF-7 at the S phase. Derivative **74** demonstrated a several-fold elevation of Bax levels (Bax level = 468.60 pg/mL) compared to the control (Bax level = 60.80 pg/mL), whereas Bcl-2 levels were downregulated to half (Bcl-2 level = 5.00 ng/mL) relative to the control (Bcl-2 level = 12.04 ng/mL). Derivative **74** exhibited a notable affinity for T790 M EGFR, with a docking score of −10.45 kcal/mol. It demonstrated hydrogen bonding with Met790, along with several hydrophobic interactions including Lys745, Val726, and Gly796 residues. Conversely, derivative **74** exhibited a commendable affinity for the HER2 active site, achieving a docking score of −10.50 kcal/mol. The NH of the sulfonamide group established a hydrogen bond with Thr862, whereas the bromine atom at the para position made a weak hydrogen binding with Asp845. Additionally, hydrophobic contacts were established between imidazole, pyrimidine, and substituted 2-phenyl rings with Cys805, Thr798, and Gly729, respectively (Figure 40).

**Figure 41:**
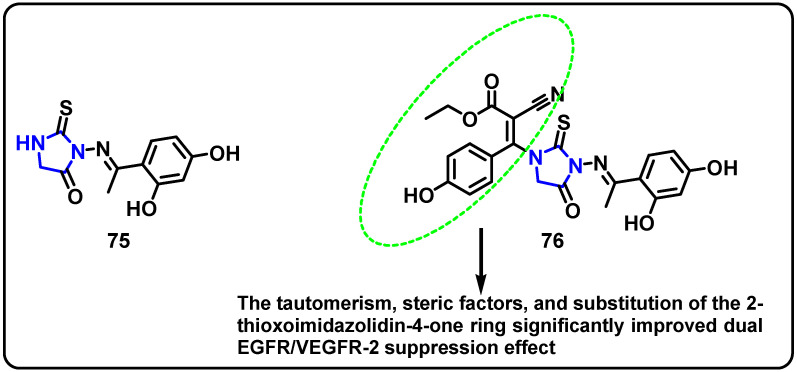
Structures of 2- thioxoimidazolidin-4-one derivatives **75** and **76** as multi-targeting tyrosine kinase inhibitors.

Mourad et al. [139] demonstrated the synthesis and inhibitory evaluation of novel 2-thioxoimidazolidin-4-one derivatives targeting EGFR and VEGFR-2. Among the screened analogs, derivatives **75** and **76** presented exceptional inhibitory effects against EGFR and VEGFR-2 (EGFR, IC_50_ = 23.16 and 11.39 nM, respectively; VEGFR-2, IC_50_ = 25.14 and 19.78 nM, respectively), in contrast to erlotinib and sorafenib (IC_50_ = 28.07 and 35.62 nM, respectively). Moreover, derivatives **75** and **76** showed significant cytotoxic activity against MCF-7, HepG2, and A549 cell lines, with IC_50_ equal to 2.26, 5.18, and 3.14 µM, respectively, for derivative **75** and IC_50_ values of 1.63, 2.44, and 1.27 µM, respectively, for derivative **76**. Additionally, derivatives **75** and **76** exhibited cell accumulation at the pre-G1 phase and induced cell cycle arrest at the G2/M phase in MCF-7 cells. As well, both derivatives displayed significant apoptotic effects mediated by the upregulation of pro-apoptotic Bax, downregulation of anti-apoptotic Bcl-2, and a notable increase in levels of p53, caspase-7, and caspase-9. The docking at the EGFR active site indicated that derivative **76** exhibited the greatest docking score of -6.04 kcal/mol. Notably, both derivatives **75** and **76** participate in hydrogen bonding with the free hydroxyl groups and Met 793. Concerning derivative **76**, the CN group participated in a hydrogen bond with Arg 841, while the C=O group was involved in a water-mediated hydrogen bond with the amino acids Asp 800, Cys 797, and Gly 796. Simultaneously, both derivatives **75** and **76** established π-H interactions with Leu718. Conversely, derivative **76** exhibited the highest docking score of –8.16 kcal/mol within the active region of VEGFR-2 kinase. Derivative **76** established three hydrogen bonds with the amino acids Lys868 and Asp1046. Additionally, a π-H interaction is established between the 4-hydroxyphenyl ring and Glu885. Furthermore, derivative **75** engages in three hydrogen bonding contacts with Phe1047, Cys919, and Asp1046. Derivative **76** exhibits eminent dual inhibitory properties against EGFR and VEGFR-2 due to keto-enol tautomerism between the 4-hydroxyphenyl and 2,4-dihydroxy groups, alongside the influence of the cyano group, sulfur atom, and carbonyl group from the 2-thioxoimidazolidin-4-one motif, which favorably impacts its binding interactions with critical amino acids in the ATP binding sites of both receptors. The tautomerism, steric considerations, and substitution of the 2-thioxoimidazolidin-4-one ring dramatically enhanced the dual suppression impact on EGFR and VEGFR-2 (Figure 41).

**Scheme 7:**
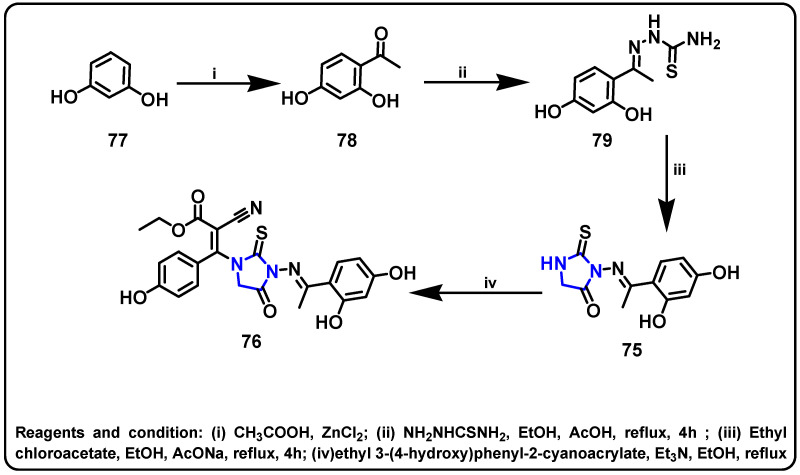
The synthesis of 2-thioxoimidazolidin-4-one derivatives **75** and **76**.

The preparation of novel 2-thioxoimidazolidin-4-one derivatives **75** and **76** were accomplished as described in Scheme 7.

**Figure 42:**
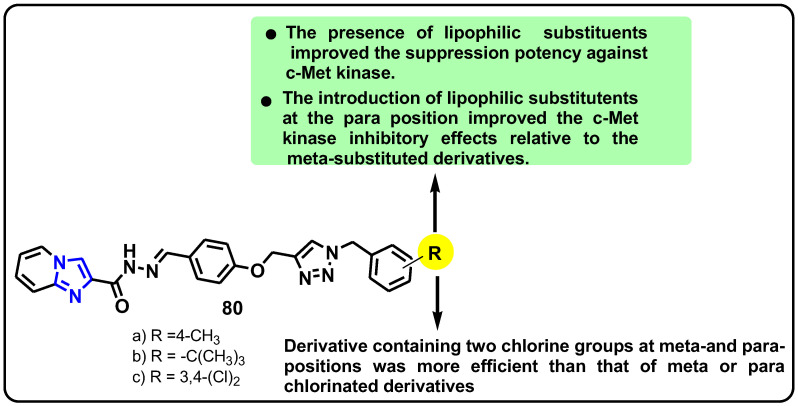
Structures of imidazo [1,2-*a*] pyridine derivatives **80a**–**c** as multi-targeting tyrosine kinase inhibitors.

New imidazopyridine derivatives bearing 1,2,3-triazole ring **80a**–**c** stood out as significant inhibitors versus both cMET and FLT-3 kinases. They suppressed c-Met at 25 μM by 55.3, 53.0, and 51.3%, respectively. They also significantly inhibited FLT-3 at 25 μM by 71, 50, and 64%, respectively. Moreover, derivative **80b** displayed anti-proliferative efficacy toward lung (EBC-1) and pancreatic cancer cells (AsPc-1 and Suit-2) that overexpress the c-MET receptor, with IC_50_ values of 3.2, 3.0, and 3.9 µM, respectively. Concerning c-Met kinase suppression, the SAR analysis demonstrated that there is a significant correlation between the nature and location of the substituent on the phenyl ring linked to the triazole moiety and the inhibitory activities of c-Met kinase. The lipophilic characteristics of the substituents evidently enhanced the inhibitory efficacy of the synthesized candidates toward c-Met kinase. The derivative with a benzyl ring exhibited minimal activity, while derivatives **80a** and **80b,** with *p*-methyl benzyl and *p*-tertiary butyl benzyl rings, respectively, demonstrated a c-Met suppression effect above 50% at a concentration of 25 μM. In addition, derivative **80c**, containing two chlorine substituents at the 3rd and 4th positions of the benzyl molecule, had a 51.3% inhibitory effect on c-Met kinase, surpassing the efficacy of either *meta* or *para* chlorinated derivatives. Additionally, it can be inferred that the incorporation of lipophilic substituents at the para position of the benzyl ring enhanced the c-Met kinase inhibitory effects compared to the *meta*-substituted derivatives. Derivative **80b**’s binding mechanism with c-Met exhibited two hydrogen bonds with Lys1110 and Met1160. The triazole ring interacts with Lys1110, Met1131, Phe1223, and Leu1157. Furthermore, pi interactions were observed between the phenyl moiety of the methoxy phenyl linker and Ala1108, Met1211, and Val1092. The *p*-tertiary butyl benzyl moiety infiltrated the posterior hydrophobic area, establishing pi–alkyl interactions with Met1131 (benzyl) and Phe1200 (tertiary butyl) (Figure 42) [140].

#### 2.6.2. Benzimidazole-Based Derivatives as Multi-Targeting Tyrosine Kinase Inhibitors

**Figure 43:**
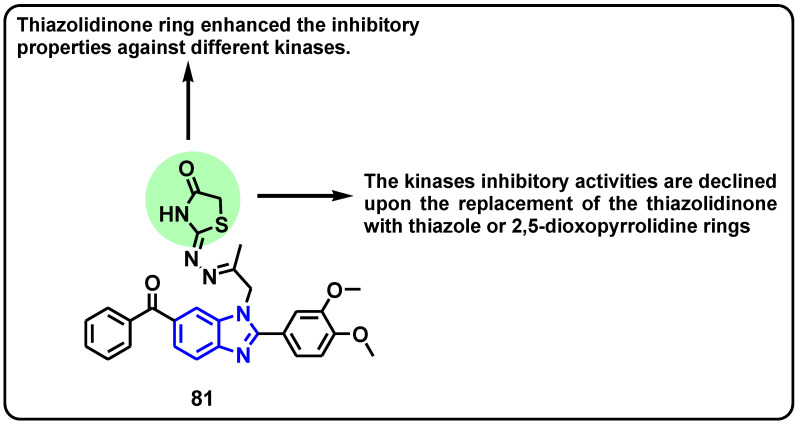
Structure of benzimidazole–thiazolidinone hybrid **81** as multi-targeting tyrosine kinase inhibitor.

In 2020, the benzimidazole–thiazolidinone hybrid **81** emerged as eminent dual inhibitor of EGFR and HER2, with IC_50_ equal to 0.109 and 0.19 µM, respectively, relative to Erlotinib, which has IC_50_ = 0.079 and 1.23 µM, respectively. The thiazolidinone derivative **81** (IC_50_ = 21.61 µM) exhibited an efficacy approximately 3.8-fold greater than that of Erlotinib (IC_50_ = 83.11 µM) in targeting PDGFR-β. In addition, it demonstrated a 1.8-fold greater efficacy than Erlotinib against VEGFR-2 (IC_50_; 69.62 µM, IC_50_ Erlotinib; 124.7 µM). The biological data indicated that the conjugation of benzimidazole core with the thiazolidinone ring reinforced the inhibitory properties against different kinases. Otherwise, the kinases-suppression effectiveness declined upon the replacement of the thiazolidinone with thiazole or 2,5-dioxopyrrolidine rings. Moreover, derivative **81** demonstrated promising cytotoxic efficiency against the Hela cell line (IC_50_ = 1.44 µM), which was higher than that exerted by Doxorubicin (IC_50_ = 2.05 µM). Compound **81** stimulated apoptosis in HeLa cells and arrested cell cycle progression at the G2/M stage. Docking research disclosed that the most efficient HER2 inhibitor **81** exhibited optimal fitting inside the active site of HER2, achieving the greatest energy score of −9.7 kcal/mol, relative to Erlotinib (energy score = −5.8 kcal/mol). Two hydrogen bonds were identified between the carbonyl and NH groups of the thiazolidine ring with Asn 850 and Asp 863, respectively. A pi–pi contact was established between the phenyl ring and Phe 864 (Figure 43) [141].

**Figure 44:**
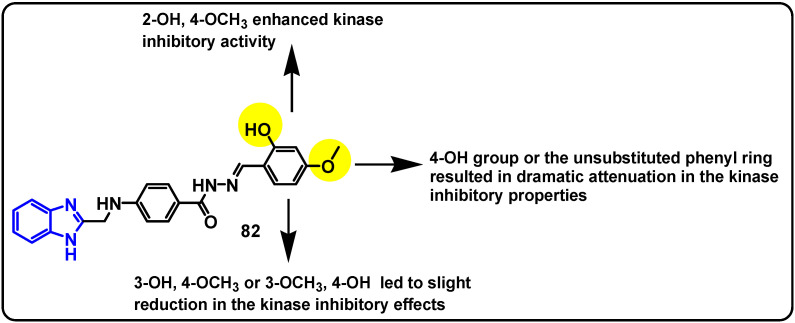
Structure of benzimidazole–hydrazone derivative **82** as multi-targeting tyrosine kinase inhibitor.

A new benzimidazole–hydrazone derivative **82** displayed promising dual inhibitory effectiveness toward EGFR and HER2, with IC_50_ = 0.091 and 0.029 µM, respectively, relative to Erlotinib and Lapatinib (IC_50_ = 0.054 and 0.034 µM, respectively). The SAR investigation demonstrated that the characteristics and placement of substituents on the phenyl ring significantly influence kinase inhibitory actions. Derivative **82**, featuring 2-OH and 4-OCH_3_, exhibited the most dual EGFR/HER2 inhibitory effectiveness. In addition, slight reductions in the kinase inhibitory effects were observed upon the change of the position of the OH and OCH_3_ groups, such as in derivatives including 3-OH, 4-OCH_3_, 3- OCH_3_, or 4-OH. Otherwise, dramatic attenuation in inhibitory properties occurred upon the substitution of the phenyl moiety with a 4-OH group or an unsubstituted phenyl ring. Furthermore, it demonstrated significant anticancer efficacy toward HCT-116, HepG-2, and MCF-7 (IC_50_ = 14.26, 10.83, and 13.26 µM, respectively), surpassing that of Sunitinib (IC_50_ = 17.91, 8.38, and 24.06 µM, respectively). Interestingly, compound **82** exhibited a weak cytotoxic effect on normal cell lines (WI-38) with an IC_50_ of 88.23 µM, in contrast to Sunitinib, which had an IC_50_ of 55.63 µM. Compound **82** triggered apoptosis in HepG-2 cells through the activation of caspase-3, upregulation of Bax, downregulation of Bcl-2, and inhibition of the cell cycle in the G0-G1 phase. The docking investigation of derivative **82** within the active pocket of EGFR revealed the establishment of a hydrogen connection between the hydroxy group and Thr766, besides hydrophobic interactions with Val702, Leu820, Lys721, Leu764, and Leu694. Conversely, derivative **82** was docked into the active region of the HER2 enzyme, where it was stabilized by a single hydrogen bond with Ser783, in addition to hydrophobic contacts with Leu785, Leu800, Lys753, Val734, and Leu852 (Figure 44) [142].

**Figure 45:**
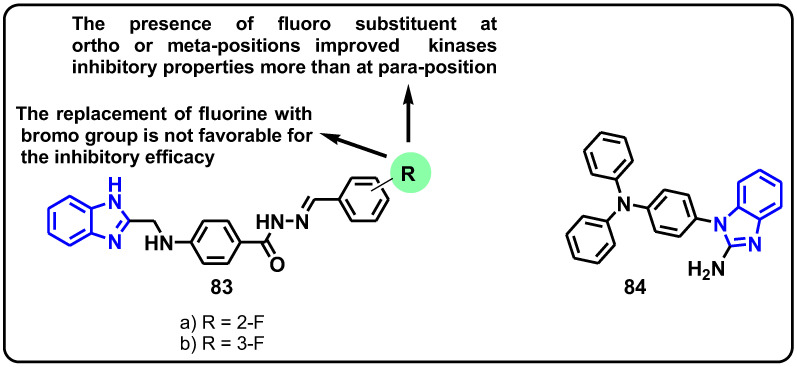
Structures of benzimidazole–benzylidenebenzohydrazide hybrids **83a**,**b** and benzimidazole–triphenylamine hybrid **84** as multi-targeting tyrosine kinase inhibitors.

Furthermore, Mirgany and coworkers [143] detailed the production and inhibitory evaluation of novel benzimidazole–benzylidenebenzohydrazide hybrids targeting several kinases. Among the examined hybrids, derivatives **83a** and **83b** exhibited promising suppression efficacy toward varied tyrosine kinase receptors, namely HER2, EGFR, and VEGFR-2, with IC_50_ equal to 23.2, 73.2, and 194.5 nM for **83a** and 28.3, 30.1, and 172.2 nM for **83b**, respectively. A SAR study indicated that the existence of a fluoro substituent on the benzylidene moiety at *ortho* or *meta* positions improved the receptor tyrosine kinases inhibitory properties more effectively than a fluoro substituent at the *para* position, while the replacement of fluorine with a bromo group is detrimental to inhibitory effectiveness. Moreover, derivative **83b** demonstrated outstanding anti-proliferative efficacy, evidenced by substantially lower IC_50_ values in diverse cancer cell lines (MCF-7; IC_50_ = 11.64 μM, HepG2; I_C50_ = 9.39 μM, HCT-116; IC_50_ = 13.44 μM) in contrast to WI-38 cells (IC_50_ = 56.46 μM), signifying pronounced selectivity towards cancer cells. Additionally, compound **83b** triggered a significant cell cycle arrest at the G1 phase in HepG2 cells. This was followed by an overexpression of caspase-3 and Bax (388.497 and 513.731 Pg/mL, respectively) and a downregulation of Bcl-2 (2.073 Pg/mL). The docking analysis of derivative **83b** within the EGFR active site revealed the establishment of H-bond interactions facilitated by water molecules with the Thr830 and Thr766 residues. Furthermore, the nitrogen atom of the imidazole moiety participated in a hydrogen bond with Asp831, while another hydrogen bond was seen between the carbonyl oxygen and Met769. Furthermore, a cation-π connection was established between the fluorine atom and Lys704. Concerning the Her2 active site, derivative **83b** established hydrogen bonding with Asp863, Lys753, and Met501, which were maintained by water molecules. Additionally, Leu852 and Leu726 participate in hydrophobic interactions with the phenyl ring of the benzylidene group. Furthermore, a benzimidazole–triphenylamine hybrid **84** emerged as remarkable anticancer candidate against MCF-7, A549, HepG2, and SPC-A-1 cancer cells with IC_50_ values of 15.61, 26.05, 17.60, and 14.48 µM, respectively. Interestingly, derivative **84** revealed prominent efficiency against highly aggressive small-cell lung cancer cells H446 (IC_50_ = 24.85 µM). Derivative **84** has a slightly greater binding affinity (−5.9 kcal/mol) than imatinib (−5.5 kcal/mol) towards mutated PDGFRα. Additionally, its affinity (−5.6 kcal/mol) is comparable to Erlotinib (−6.9 kcal/mol) against mutant EGFR T790M/L858R. The in silico ADME study revealed the ideal nature of **84** as a druggable anticancer candidate for further development (Figure 45) [144].

**Figure 46:**
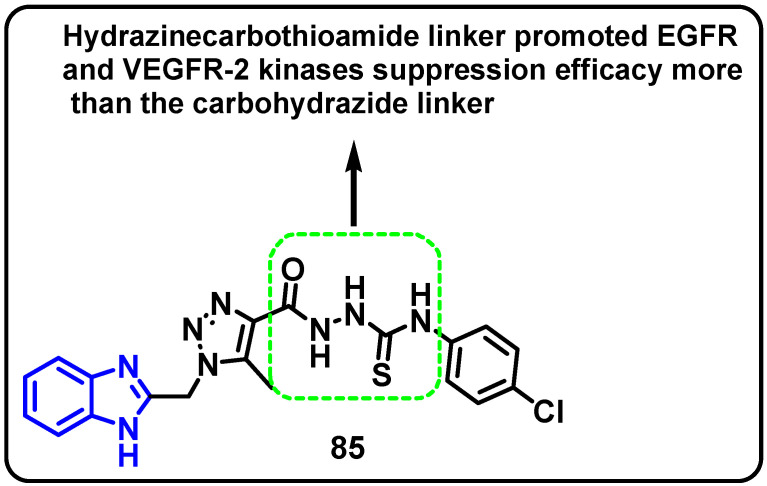
Structure of benzimidazole–triazole hybrid **85** as multi-targeting tyrosine kinase inhibitor.

A new benzimidazole–triazole hybrid **85** emerged as an inhibitor for both EGFR and VEGFR-2, with strong values of IC_50_ equal to 0.086 and 0.107 µM, respectively. SAR analysis indicated that the existence of a hydrazinecarbothioamide linker promoted EGFR and VEGFR-2 kinases suppression efficacy more than the presence of a carbohydrazide linker. In addition, derivative **85** demonstrated prominent cytotoxic efficiency on HeLa, HCT-116, HepG-2, and MCF-7 cancer cells (IC_50_ = 3.87, 8.34, 7.68, and 6.81 µM, respectively). Furthermore, derivative **85** induced apoptosis and arrested growth of HepG-2 cells at the S phase. Moreover, the DNA binding results revealed that derivative **85** exhibited comparable activity (IC_50_ = 33.17 µM) as Dox (IC_50_ = 31.54 µM). Additionally, derivative **85** presented fit binding with the key active sites of both EGFR and VEFGR-2 kinases (Figure 46) [145].

**Scheme 8:**
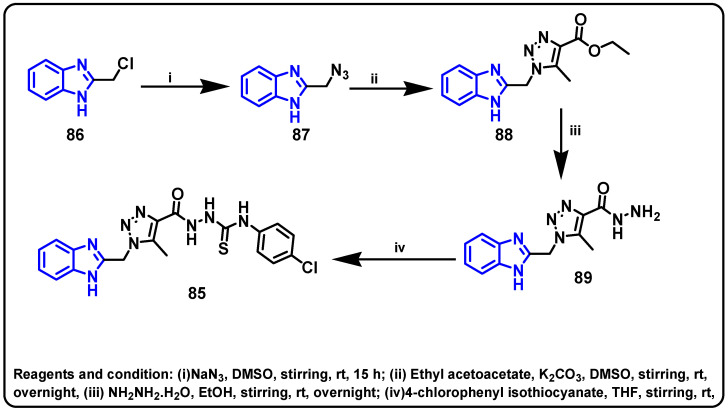
The synthesis of benzimidazole–triazole hybrid **85**.

2-Chloro methyl benzimidazole **86** was reacted with sodium azide to afford the corresponding azide **87**, which underwent condensation reaction with ethyl acetoacetate to obtain the ester derivative **88**. The hydrazide derivative **89** was achieved via the reaction of the ester derivative **88** with hydrazine hydrate. The new benzimidazole–triazole hybrid **85** was obtained by reacting the hydrazide **89** with the 4-chlorophenyl isothiocyanate (Scheme 8).

**Figure 47:**
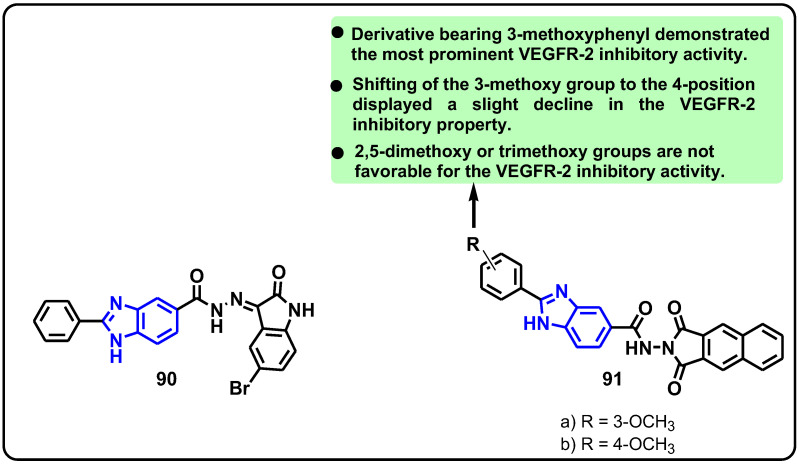
Structures of benzimidazole–oxindole conjugate **90** and benzimidazole–dioxobenzoisoindoline hybrids **91a**,**b** as multi-targeting tyrosine kinase inhibitors.

A new benzimidazole–oxindole conjugate **90** emerged as a promising dual inhibitor of VEGFR-2 and FGFR-1, with IC_50_ equal to 0.18 and 1.65 µM, respectively, in contrast to sorafenib, which demonstrated IC_50_ values of 0.10 and 0.58 µM, respectively. Derivative **90** had the most favorable GI_50_ range across several subpanels, with a GI_50_ levels at 1.23–3.38 μM in melanoma cell lines. Moreover, derivative **90** displayed an effective value for GI_50_ = 1.53–3.72 μM, 1.38–3.00 μM, and 1.07–3.55 μM toward ovarian, NSCLC, and breast cancer cells, respectively. Moreover, compound **90** disclosed a potent GI_50_ with values 0.42–2.11 μM on CNS cancer cells. Surprisingly, derivative **90** exhibited a notable safety profile on HSF, namely, the normal human skin fibroblast cell line (IC_50_ > 100 μM). Also, derivative **90** suppressed mRNA expression of FGFR-1 and VEGFR-2 and disrupted the Notch1/TGFβ1 pathway in tumor cells. Additionally, derivative **90** presented strong binding affinity with energy values of −15.18 kcal/mol (VEGFR-2) and −17.00 kcal/mol (FGFR-1). H-bond interactions were detected between the nitrogen atom of benzimidazole and the glutamic acid residues Glu885 and Glu531 of the αC helix, as well as with aspartic acid residues Asp1046 and Asp641 of the conserved DFG motif in VEGFR-2 and FGFR-1, respectively. Furthermore, hydrophobic interactions were established between the benzimidazole ring and the hydrophobic side chains of Val899, Phe1047, Val916, Cys1045, Lys868, and Val848 in VEGFR-2, as well as Lys514, Phe642, Ala640, Ile545, Val492, and Val561 in FGFR-1 (Figure 47) [146]. Furthermore, new benzimidazole–dioxobenzoisoindoline hybrids **91a** and **91b** were discovered as remarkable dual VEGFR-2 and FGFR-1 inhibitors (% inhibition = 87.61 and 80.69%, respectively, for VEGFR-2 and 84.20 and 76.83%, respectively, for FGFR-1 at 10 µM). Concerning the VEGFR-2 inhibitory effect, SAR analysis indicated that derivative **91a** bearing 3-methoxyphenyl connected to the benzimidazole scaffold demonstrated the most prominent inhibitory activity. Shifting of the 3-OCH_3_ group to the 4th position to afford **91b** displayed a slight decline in the inhibitory property. On the other hand, a drastic reduction in the inhibitory effectiveness was exerted by the replacement of 3-methoxy with the 2,5-dimethoxy group. Whereas total loss of the suppression effect was observed by exchanging 3-methoxy by the 3,4,5-trimethoxy group. Moreover, derivative **91b** had the most pronounced cytotoxic effect on MCF-7 cells, with an IC_50_ of 5.03 µM, in contrast to sorafenib, which had an IC_50_ of 3.24 µM. Derivative **91b** triggered early and late apoptosis in MCF-7 cells and ceased the cell cycle at the G2/M phase. The benzimidazole–dioxobenzoisoindoline conjugate **91b** was docked in the binding active sites of FGFR-1and VEGFR-2, yielding binding energy scores of −9.51 and−10.70 kcal/mol, respectively. The dioxobenzoisoindoline moiety is located in the ATP binding site, where one carbonyl (C=O) group forms H-bonds with the Ala564 and Cys919 residues in the binding sites of FGFR-1 and VEGFR-2, respectively. Furthermore, Val848, Leu840, Leu1035, Phe918, Ala866, and Cys919 in VEGFR-2 participate in hydrophobic connections with the dioxobenzoisoindoline molecule. Ala512, Leu484, Leu630, and Val492 amino acids in FGFR-1 engage in hydrophobic interactions with the dioxobenzoisoindoline moiety. In addition, the Glu531 and Glu885 residues in FGFR-1 and VEGFR-2, respectively, participate in hydrogen bonding with the NH of the benzimidazole core. The benzimidazole scaffold formed hydrophobic interactions with Val916, Leu889, Lys868, Cys1045, and Val899, the amino acids in the gate region of VEGFR-2, as well as with Val561, Lys514, Ile545, Met535, Ala640, and Leu614 in FGFR-1 (Figure 47) [147].

**Figure 48:**
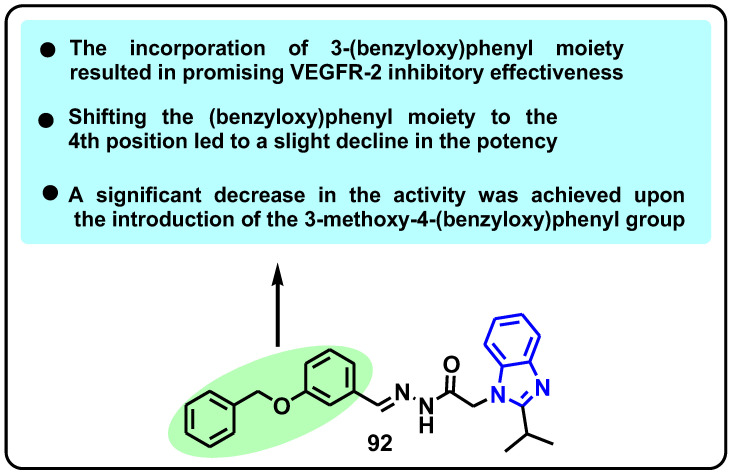
Structure of 1,2-disubstituted benzimidazole derivative **92** as multi-targeting tyrosine kinase inhibitor.

A new 1,2-disubstituted benzimidazole derivative **92** was reported as a promising inhibitor of PDGFR-β, FGFR-1, and VEGFR-2, exhibiting IC_50_ values of 0.05, 0.11, and 0.11, µM, respectively. The inclusion of the 3-(benzyloxy)phenyl moiety demonstrated significant inhibitory efficacy toward VEGFR-2. Shifting the (benzyloxy)phenyl moiety to the 4th position resulted in a little reduction in efficacy. Nonetheless, a substantial reduction in activity was attained with the incorporation of the 3-methoxy-4-(benzyloxy)phenyl group. Furthermore, derivative **92** had a significant cytotoxic effect against HepG2 cells, with an IC_50_ of 1.98 µM, compared to sorafenib, which has an IC_50_ of 10.99 μM. Furthermore, derivative **92** elicited a dose-dependent apoptotic response and inhibited cell cycle progression at the G2/M phase. The candidate **92** effectively occupied the VEGFR-2 binding site with a docking score of −14.31 kcal/mol. The hydrazide–hydrazone moiety participates in hydrogen bonding interactions with Glu885 of the αC helix and Asp1046 in the conserved DFG motif. Additionally, the 2-substituted benzimidazole molecule is situated within the allosteric hydrophobic pocket, establishing hydrophobic interactions with Ile1044, Ile888, Val899, Val898, Ile892, Leu1019, and Leu889. The extension on the distal phenyl moiety exhibited hydrophobic interactions with the amino acids Leu1035, Phe918, Leu840, Phe1047, and Cys919 (Figure 48) [148].

**Figure 49:**
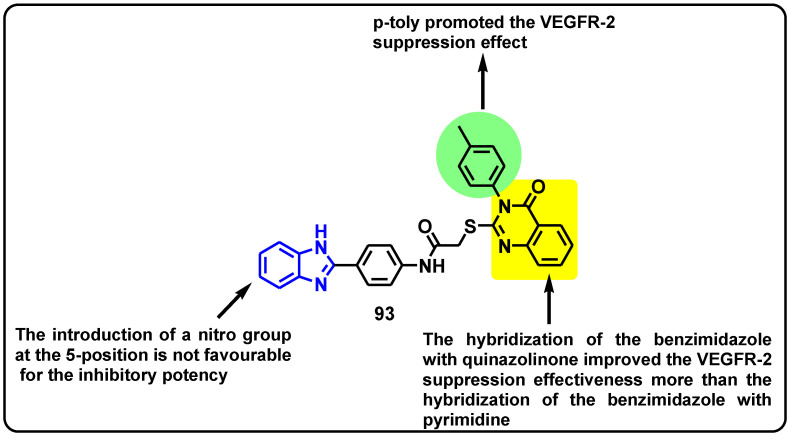
Structure of benzimidazole–quinazolinone hybrid **93** as multi-targeting tyrosine kinase inhibitor.

Ali and colleagues [149] detailed the synthesis and evaluation of the inhibitory efficacy of novel benzimidazole–pyrimidine and benzimidazole–quinazolinone hybrids against various tyrosine kinases. Among the examined hybrids, the benzimidazole–quinazolinone hybrid **93** emerged as promising a multi-kinase inhibitor targeting FLT-3, PDGFR-β, and VEGFR-2, with IC_50_ values of 0.13, 0.03, and 6.14 µM, respectively, relative to sorafenib (IC_50_ = 0.06, 0.04, and 2.88 μM, respectively). SAR analysis displayed that the hybridization of benzimidazole with quinazolinone improved the efficacy of VEGFR-2 suppression more than the hybridization of benzimidazole with pyrimidine. Additionally, the incorporation of a NO_2_ group at position-5 of the benzimidazole moiety does not enhance inhibitory efficacy. The substitution of quinazolinone with methyl, phenyl, ethyl, and p-chlorophenyl groups is unfavorable for VEGFR-2 inhibitory activity. Otherwise, the substitution of quinazolinone with p-tolyl or 4-methoxyphenyl enhanced the VEGFR-2 inhibitory actions. At the VEGFR-2 binding site, derivative **93** had a favorable docking score that equaled −14.82 kcal/mol, in comparison to sorafenib, which had a docking value of −15.19 kcal/mol. The NH group of the benzimidazole scaffold participates in hydrogen bonding with Glu885. The benzimidazole scaffold engaged in hydrophobic interactions with the amino acids Leu889 and Val899. The quinazolinone participates in hydrophobic interactions with Leu1035, Leu840, Cys919, Phe1047, and Phe918 (Figure 49).

## 3. Discussion and Perspectives

Cancer is a lethal illness, and the mortality rate from different cancer kinds rises year. Receptor tyrosine kinases (RTKs) facilitate intercellular communication and govern intricate biological processes like cell proliferation, differentiation, motility, and metabolism. The 58 identified human receptor tyrosine kinases (RTKs) possess a solitary transmembrane helix, an extracellular ligand-binding domain, an intracellular segment featuring a carboxyl-terminal (C-) tail, a tyrosine kinase domain (TKD), and a membrane-associated regulation region. Disruption of receptor tyrosine kinase signaling is associated with various illnesses, including cancer [11]. The benzimidazole ring structure is analogous to the guanine and adenine nucleotides constituting DNA and RNA, hence enhancing its anticancer properties [17,18]. Numerous investigations employ benzimidazole ring hybrids with heterocyclic rings to ascertain molecules that elicit certain biological effects. The bioavailability, stability, and extensive receptor action of benzimidazole-based compounds render them appealing candidates for anticancer therapeutics. Numerous therapeutically authorized anticancer agents are derived from the benzimidazole ring structure, as indicated in Table 1 with the promising values of IC_50_ against various types of tyrosine kinase receptors. The hybridization of imidazole or benzimidazole with other heterocyclic moieties enhances their activity [53]. In numerous instances, the inclusion of electron-donating groups, such as methoxy groups, enhances their activity, whereas electron-withdrawing groups, in certain cases, diminish their efficacy. Furthermore, the activity of the imidazole derivatives was augmented when halogen atoms such as fluorine, bromine, or chlorine were included into their structures [53,54,61]. Imidazole and benzimidazoles stimulated apoptosis through activating Bax, caspase-3, and caspase-8 [65]. The nitrogen atoms in the imidazole core are crucial for interacting with the active site of each kinase protein by forming various types of hydrogen bonds. Furthermore, imidazole and benzimidazole derivatives can occupy the coordination site of ATP; it forms hydrogen bonds with the glutamic acid (Glu) residue in the active site of the Abl kinase protein; it can coordinate metal ions in competition with histidine amino acid; the imidazole ring also allows it to participate in van der Waals interactions and pi–pi stacking within the binding pocket, contributing to the overall binding affinity and stability of the inhibitor–kinase complex. From this review article, scientists can design new systems carrying an imidazole moiety hydride with the active species to improve the activity against several types of kinase receptors.

Table 1 summarizes some imidazole and benzimidazole-based derivatives targeting various receptor tyrosine kinases, displaying their IC_50_ values.

## 4. Conclusions

Studies on innovative cancer therapies have evolved from an era of toxic, non-specific drugs to more effective, targeted options. The primary drawbacks of chemotherapeutic agents are their lack of selectivity and the consequent toxicity. Thus, substantial evidence exists to bolster the enhancement of targeted therapy with localized action in combating this deadly condition. Comprehensive research in pharmaceutical discovery and an understanding of cancer biology led to the thorough exploration of imidazole pharmacophore-based compounds as targeted kinase therapies for cancer. The current review focused on imidazole and benzimidazole derivatives as inhibitors for various receptor tyrosine kinase targets, such as EGFR, c-Met, VEGFR, FLT3, and FGFR. In this context, various receptor tyrosine kinase-targeting imidazoles and benzimidazoles are examined with a thorough explanation of their SARs. This data will aid in producing more powerful and selective imidazole and benzimidazole derivatives as kinase inhibitors through further structural alterations of various imidazole and benzimidazole-based derivatives. Additionally, the combination of imidazole and benzimidazole frameworks with other active heterocyclic derivatives play a prominent role in discovering new lead anticancer candidates with enhanced efficacy against receptor tyrosine kinases. The versatility and utility of the imidazole and benzimidazole moieties in modern pharmaceutical chemistry are demonstrated by the several synthesized derivatives displayed in this review, which have potencies that are comparable or greater than that of the reference drugs. We anticipate that our assessment will assist pharmaceutical experts, including medicinal chemists, in creating new anticancer drugs that are safer and more effective targeting receptor tyrosine kinases.

## Figures and Tables

**Table 1:** Examples of imidazole and benzimidazole-based derivatives as receptor tyrosine kinase inhibitors.

Structure	Target Kinase	IC_50_	Reference
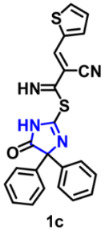	EGFR	0.137 µM	[45]
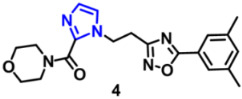	EGFR	0.47 μM	[47]
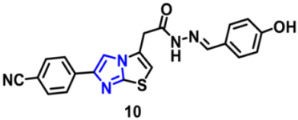	EGFR	9.11 µM	[50]
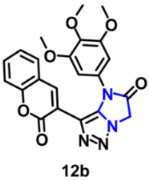	EGFR	0.367 µM	[52]
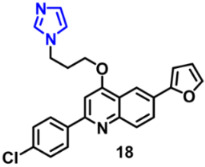	EGFR	33.65 nM	[57]
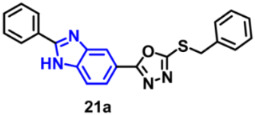	EGFR	61 nM	[60]
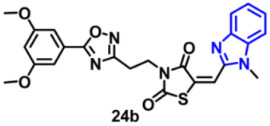	EGFR	0.23 µM	[63]
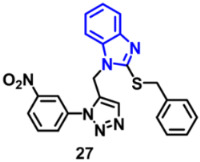	EGFR	73 nM	[65]
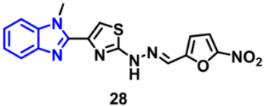	EGFR	71.67 nM	[66]
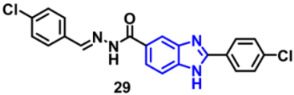	EGFR	0.09 µM	[67]
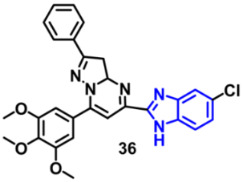	EGFR	0.29 µM	[69]
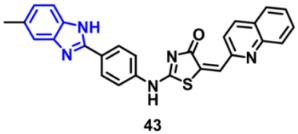	EGFR	0.47 μM	[71]
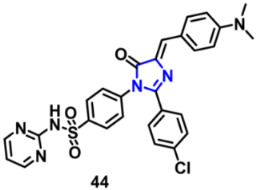	VEGFR-2	0.07 µM	[79]
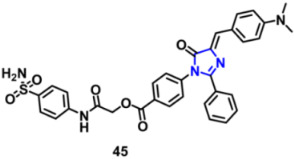	VEGFR-2	0.02 µM	[79]
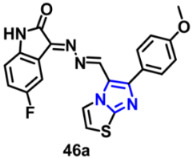	VEGFR-2	0.22 µM	[80]
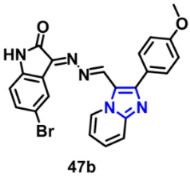	VEGFR-2	0.28 µM	[80]
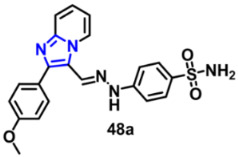	VEGFR-2	0.576 µM	[81]
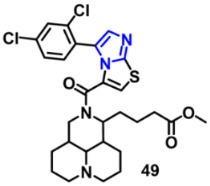	VEGFR-2	3.09 μM	[82]
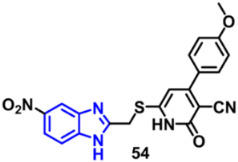	VEGFR-2	2.83 μM	[84]
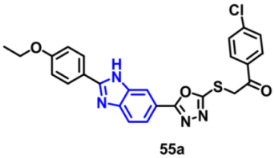	VEGFR-2	0.475 μM	[85]
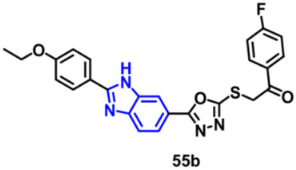	VEGFR-2	0.618 µM	[85]
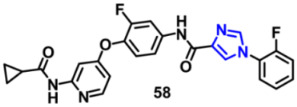	c-Met	0.012 μM	[97]
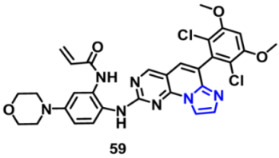	FGFR1, 2, and 4	8, 4, and 3.8 nM, respectively	[118]
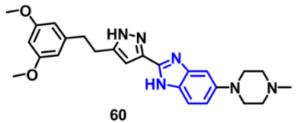	FGFR (1-3)	0.75, 0.50, and 3.05 nM, respectively	[119]
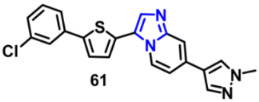	FLT3	0.053 µM	[130]
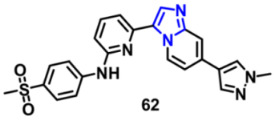	FLT3	7.94 nM	[131]

## Data Availability

No new data were created or analyzed in this study. Data sharing is not applicable to this article.
